# European white paper: oropharyngeal dysphagia in head and neck cancer

**DOI:** 10.1007/s00405-020-06507-5

**Published:** 2020-12-19

**Authors:** Laura W. J. Baijens, Margaret Walshe, Leena-Maija Aaltonen, Christoph Arens, Reinie Cordier, Patrick Cras, Lise Crevier-Buchman, Chris Curtis, Wojciech Golusinski, Roganie Govender, Jesper Grau Eriksen, Kevin Hansen, Kate Heathcote, Markus M. Hess, Sefik Hosal, Jens Peter Klussmann, C. René Leemans, Denise MacCarthy, Beatrice Manduchi, Jean-Paul Marie, Reza Nouraei, Claire Parkes, Christina Pflug, Walmari Pilz, Julie Regan, Nathalie Rommel, Antonio Schindler, Annemie M. W. J. Schols, Renee Speyer, Giovanni Succo, Irene Wessel, Anna C. H. Willemsen, Taner Yilmaz, Pere Clavé

**Affiliations:** 1 Department of Otorhinolaryngology, Head and Neck Surgery, Maastricht University Medical Center, Maastricht, The Netherlands; 2 GROW School for Oncology and Developmental Biology, Maastricht University Medical Center, Maastricht, The Netherlands; 3 Department of Clinical Speech and Language Studies, Trinity College Dublin, Dublin, Ireland; 4 Department of Otorhinolaryngology, Head and Neck Surgery, Helsinki University Hospital, University of Helsinki, Helsinki, Finland; 5 Department of Otorhinolaryngology, Head and Neck Surgery, University Hospital Magdeburg, Otto-von-Guericke University, Magdeburg, Germany; 6 Department of Special Needs Education, University of Oslo, Oslo, Norway; 7 School of Occupational Therapy, Social Work and Speech Pathology, Curtin University, Perth, Australia; 8 Department of Neurology, Born Bunge Institute, Antwerp University Hospital, University of Antwerp, Antwerp, Belgium; 9 Voice, Speech, Swallowing Lab, Department of Otorhinolaryngology, Head and Neck Surgery, University Hospital UVSQ and Research lab CNRS-UMR7018, Hôpital Foch, Suresnes, France; 10 Swallows Head and Neck Cancer Charity, Blackpool, UK; 11 Department of Head and Neck Surgery, The Greater Poland Cancer Centre, Poznan University of Medical Sciences, Poznan, Poland; 12 Head and Neck Cancer Centre, University College London Hospital, London, UK; 13 Department of Experimental Clinical Oncology, Aarhus University Hospital, Aarhus, Denmark; 14 Department of Otorhinolaryngology, Head and Neck Surgery, Medical Faculty, University of Cologne, Cologne, Germany; 15 Robert White Centre for Airway, Voice and Swallow, Poole Hospital NHS Foundation Trust, Dorset, UK; 16 Deutsche Stimmklinik, Hamburg, Germany; 17 Departement of Voice, Speech and Hearing Disorders, University Medical Center Hamburg-Eppendorf, Hamburg, Germany; 18 Department of Otolaryngology, Head and Neck Surgery, Faculty of Medicine, Atılım University, Medicana International Ankara, Ankara, Turkey; 19 Department of Otolaryngology, Head and Neck Surgery, Amsterdam University Medical Centres, Vrije Universiteit, Amsterdam, The Netherlands; 20 Cancer Center Amsterdam, Amsterdam University Medical Centres, Vrije Universiteit, Amsterdam, The Netherlands; 21 Division of Restorative Dentistry and Periodontology, Faculty of Health Sciences, Trinity College Dublin, Dublin Dental University Hospital, Dublin, Ireland; 22 Department of Otorhinolaryngology, Head and Neck Surgery, Rouen University Hospital, Rouen, France; 23 Department of Ear Nose and Throat Surgery, The Robert White Centre for Airway Voice and Swallowing, Poole Hospital NHS Foundation Trust, University of Southampton, Southampton, UK; 24 Department of Speech and Language Therapy, St. James’s Hospital, Dublin, Ireland; 25 MHeNs School for Mental Health and Neuroscience, Maastricht University Medical Center, Maastricht, The Netherlands; 26 Department Neurosciences, Experimental Otorhinolaryngology, Deglutology, University of Leuven, Leuven, Belgium; 27 Department of Biomedical and Clinical Sciences “L. Sacco”, University of Milan, Milan, Italy; 28 Department of Respiratory Medicine, NUTRIM School of Nutrition and Translational Research in Metabolism, Maastricht University Medical Center, Maastricht, The Netherlands; 29 Department of Otorhinolaryngology and Head and Neck Surgery, Leiden University Medical Centre, Leiden, The Netherlands; 30 Faculty of Health, School of Health and Social Development, Victoria, Australia; 31 Head and Neck Oncology Service, Candiolo Cancer Institute, FPO - IRCCS, Candiolo, TO Italy; 32 Department of Oncology, University of Turin, Orbassano, TO Italy; 33 Department of Otorhinolaryngology, Head and Neck Surgery and Audiology, Rigshospitalet, University of Copenhagen, Copenhagen, Denmark; 34 Division of Medical Oncology, Department of Internal Medicine, Maastricht University Medical Center, Maastricht, The Netherlands; 35 Department of Otorhinolaryngology, Head and Neck Surgery, Faculty of Medicine, Hacettepe University, Ankara, Turkey; 36 Gastrointestinal Physiology Laboratory, Hospital de Mataró, Universitat Autònoma de Barcelona, Mataró, Spain; 37 Centro de Investigación Biomédica en Red de Enfermedades Hepáticas y Digestivas (CIBERehd), Barcelona, Spain

**Keywords:** Head and neck cancer, Dysphagia, White paper, Deglutition, Swallowing

## Abstract

**Purpose:**

To develop a European White Paper document on oropharyngeal dysphagia (OD) in head and neck cancer (HNC). There are wide variations in the management of OD associated with HNC across Europe.

**Methods:**

Experts in the management of specific aspects of OD in HNC across Europe were delegated by their professional medical and multidisciplinary societies to contribute to this document. Evidence is based on **s**ystematic reviews, consensus-based position statements, and expert opinion.

**Results:**

Twenty-four sections on HNC-specific OD topics.

**Conclusion:**

This European White Paper summarizes current best practice on management of OD in HNC, providing recommendations to support patients and health professionals. The body of literature and its level of evidence on diagnostics and treatment for OD in HNC remain poor. This is in the context of an expected increase in the prevalence of OD due to HNC in the near future. Contributing factors to increased prevalence include aging of our European population (including HNC patients) and an increase in human papillomavirus (HPV) related cancer, despite the introduction of HPV vaccination in various countries. We recommend timely implementation of OD screening in HNC patients while emphasizing the need for robust scientific research on the treatment of OD in HNC. Meanwhile, its management remains a challenge for European professional associations and policymakers.

## Introduction

The state of the art and minimum standards of oropharyngeal dysphagia (OD) care will vary by country across Europe and will depend on the setting of the medical care [acute hospital, rehabilitation unit, community nursing home, speech-and-language pathologist (SLP) first-line practice, etc.], the cultural and religious background of the population, and the vision and resources available to policymakers. Given the need for a consensus across Europe for the treatment of OD in head and neck cancer (HNC), The European Society for Swallowing Disorders (ESSD) initiated an alliance with relevant European professional medical and multidisciplinary societies to write a White Paper on this topic. The purpose of this document is to inform key stakeholders including health professionals from the various disciplines involved in the management of OD associated with HNC about the state of the art with regard to the different aspects of care. The document was written by experts from the ESSD, the Confederation of European Otorhinolaryngology Head and Neck Surgery (CEORL-HNS), the European Head and Neck Society (EHNS), the Union of the European Phoniatricians (UEP), and the European Laryngological Society (ELS). It comprises 24 sections covering all aspects of head and neck oncology in a broad sense including legal and ethical issues. A concept document comprising HNC-related topics acted as a foundation for the mandated contributors to add topics or ideas from their professional perspective. Wherever possible, a section was supported by a systematic literature review and we aimed to provide an up-to-date overview of what we believe should be essential core knowledge for health professionals and what should be the minimum standard of care for OD in the HNC setting.

## Development of head and neck cancer

### Etiology, epidemiology, and survival of head and neck cancer

HNC is one of the most common malignancies in the world, with high mortality rates. HNC comprises epithelial tumors of the nasal cavity and paranasal sinuses, nasopharynx, hypopharynx, larynx, oropharynx, oral cavity, lip, and malignant tumors of the salivary glands. More than 90% of these cancers are squamous cell carcinomas. The incidence rates of each cancer type vary according to geographic region, age, gender, and various risk factors. Globally, HNC affected approximately 686,328 individuals in 2012 [1]. The majority of these cancers are located in the oral cavity including the lip and, in the larynx, followed by the pharynx [1]. It is estimated that approximately 151,000 new patients will be affected by HNC in Europe in 2020 [2]. Tobacco and alcohol have long been considered as the main causes of HNC. It has been reported that 72% of HNCs are related to tobacco smoking and/or alcohol drinking. Although tobacco and alcohol are considered independent risk factors, within this 72% 4% are attributed to alcohol alone, 33% to tobacco, and 35% to the combined use of alcohol and tobacco [3]. HNC is seen more often in men (three to five times higher) than in women, but this gender-related prevalence differs by tumor site. Geographical variation along with the differences in prevalence between men and women have been attributed to the differences in exposure to risk factors between the two sexes [4]. However, the male to female HNC ratio has been declining over time probably due to the growing number of female smokers. On the other hand, the incidence of smoking-related cancers such as cancers of the oral cavity, larynx, and other subsites is decreasing in North America and Western Europe, probably as a result of the reduced use of tobacco products [5]. There is also an age-related difference in HNC incidence. The highest HNC incidence has been observed in patients aged 65 years and older. However, the incidence of oropharyngeal and nasopharyngeal cancer is highest between the age of 25 and 64 years [4]. This is associated with viral carcinogenesis in this age group [4]. For example, the Epstein-Barr virus (EBV) has been associated with nasopharyngeal carcinoma [6] and in recent decades human papillomavirus (HPV), usually HPV type 16 and 18, has attracted attention as a significant risk factor in the development of oropharyngeal cancer [6]. Sexual behavior is a well-recognized risk factor in the development of HPV-related HNC, in particular the lifetime number of sexual and oral sex partners [6]. Approximately one-third of all oropharyngeal cancers are HPV positive. The highest HPV prevalence is seen in tumors of the tonsil and base of the tongue. In this younger patient population tumors usually present as a small primary lesion with large metastatic lymph nodes. According to Wagner and colleagues, the prognosis of HPV-related HNC is better than HNC evoked by abuse of tobacco and alcohol [7]. The 5-year relative survival of oropharyngeal cancer increased from 37% in 1999 to 44% in 2007 [4]. The current (2019) five-year overall survival rate of HNC is approximately 40–50% [4]. Nonetheless, variations in survival statistics vary across different parts of Europe. Mortality rates are higher in Eastern Europe while Northern Europe has the highest 5-year survival rate [4]. About one-third of the HNCs present during the early stages and cure rates for these patients can reach up to 80%. In contrast, two-thirds of the patients are diagnosed in an advanced stage of the disease in which the survival rates are lower than 40%. Across Europe, the epidemiology of HNC reveals variations in tumor site, geography, age, gender, and survival rates. These differences may be attributed to different cultural habits regarding risk factors for developing HNC. Finally, syndromic and familial forms of HNC have been described [8-11]. Patients with syndromes such as xeroderma pigmentosum, Fanconi’s anemia, dyskeratosis congenita, Bloom’s syndrome, Lynch-II syndrome, *P*16 germline mutations, and ataxia-telangiectasia have a high risk of developing oral squamous cell carcinoma at younger ages and in the absence of carcinogen exposure [8–11]. HNC treatment modalities and the organization and delivery of care in multidisciplinary team (MDT) set-up are also having an effect on treatment outcome and survival rates as described in "Oncological treatment and its evolution" section.

### Vulnerable head and neck cancer population—socioeconomic disparity

As stated above, alcohol is a risk factor for developing HNC and alcohol abuse is an independent predictor of survival in HNC [12]. In a prospective study of 649 HNC patients, alcoholism and history of alcohol-related systemic health problems such as liver disease or pancreatitis were associated with increased mortality risk, whereas abstinence was associated with decreased risk of death [13]. Moreover, opioid naïve patients with a history of alcohol abuse had an increased risk for prolonged opioid use under radiotherapy (RT) [14]. Thus, it is essential to assess HNC patients’ history of alcohol consumption. As indicated earlier, tobacco is an important known risk factor for developing primary and relapsing HNC ("Etiology, epidemiology, and survival of head and neck cancer" section), patients need counseling and help to quit smoking. Some HNC units have established smoking cessation programs and support and self-help programs are available on the internet on several open access webpages. In 2014 the European Head and Neck Cancer Society published the *Make Sense Campaign*, which provides recommendations for best practices in the management of psychological needs of HNC patients [15]. Emotions and reactions can vary during the cancer patient’s ‘journey’, and health professionals should be aware of these to provide adequate psychological support (see "Psychosocial impact" section). Optimal timing for suggestions/recommendations to attend tobacco and/or alcohol withdrawal programs is during the posttreatment period [15]. However, patients are usually instructed to cease alcohol and/or tobacco consumption before HNC treatment commences.

The effects of low education, poverty, and social disadvantage are important considerations for the prognosis of HNC patients. African Americans and patients with lower socioeconomic status have a worse prognosis for HNC [16]. Dramatic disparities by ethnicity and socioeconomic status are not completely explained by demographics and comorbidity. Earlier diagnosis of HNC and timely access to surgery and adjuvant therapies could improve the outcomes for these HNC patients [16]. Neighborhood deprivation is also associated with poorer overall survival rates and an increased risk of developing second primary HNC among oropharyngeal cancer patients. Attention should therefore be paid not only to the individual but also to neighborhood-related risk factors [17]. Compared with patients with other cancer types, patients with HNC are more typically members of a minority race/ethnicity, male, poor, publicly insured, lower level of education, and with lower general medical and mental health status [18]. Attempts to improve these patients’ situation include amongst other initiatives, establishing special centers for homeless cancer patients [19]. Moreover, treatment related to gender is also possible. A study by Park and colleagues revealed that women with locoregional advanced HNC may be undertreated, and the authors supported this finding using statistical models to identify those at most risk for undertreatment [20]. In the context of oral cavity cancer, patients with mental health difficulties are considered to have a poorer prognosis when compared to those without mental health issues [21]. People with intellectual disability as well as people with dementia may have difficulties recognizing and communicating discomfort and pain during the development of HNC. In oral cavity cancer, for example, this may lead to a delayed diagnosis [22]. Poverty can affect outcomes for HNC patients. OD rehabilitation and dietary supplements are expensive, and without a proper social security system, medical care may be inaccessible to a significant number of HNC patients, as is the case in many European countries.

In developing countries, multiple factors other than poverty play a role in accessing HNC treatment. It is essential to create training programs and fellowships for physicians, nurses and other health and social care professionals both in their own countries and abroad to improve this situation [23]. In Europe, it is important to recognize that some HNC patient populations are highly vulnerable. By increasing equity of treatment access, supporting socioeconomic, racial, and gender equality and improving education and screening programs, we argue that we can reduce the risk factors for HNC, improve outcomes and lower the rate of cancer recurrence in this vulnerable population.

## Oncological treatment and its evolution

The treatment of choice for the majority of HNC patients comprises surgery and/or (chemo)radiotherapy [(C)RT]. Conservative transoral surgical procedures can offer a solution in the event of early HNC stages (T1–T2) of the oral cavity, the oropharynx, the hypopharynx, and the larynx. The progressive development of transoral robotic surgery (TORS) and transoral laser (micro)surgery (TOLS) in recent years has ensured that these techniques are used worldwide now as a single modality treatment and that the surgical results in early-stage HNC with regard to the oncological outcome are satisfactory [24–26]. The benefits of TORS and TOLS for early-stage tumors include a less invasive surgical technique, which means that the period of hospital stay is shorter and the risk of loss of function is considerably lower. It is recommended that these techniques should be carried out in well-selected centers by experienced surgeons [27]. In the case of more advanced tumors, the need for more ‘aggressive’ procedures such as open neck surgery is recognized, often leading to severe upper aerodigestive tract and aesthetic function loss depending on the tumor subsite and the tumor stage. Although the improvement of reconstructive surgical techniques, such as the use of microvascular free flaps for cancers of the oral cavity, oropharynx, hypopharynx, and sometimes larynx, or the insertion of a voice prosthesis after total laryngectomy (TLE) has led to substantial progress in functional and aesthetic results, these surgical procedures often result in speech and swallowing impairment, which should be discussed with the patient prior to surgery [28]. Surgical treatment of advanced-stage HNC is usually followed by postoperative (C)RT. It remains a matter of debate as to whether postoperative RT or CRT nullifies the beneficial functional outcome of reconstructive surgery. There are, however, clear indications that RT dose-reduction on the pharyngeal constrictor muscles may lead to a significant reduction in immediate and late swallowing morbidity [29]. Recent retrospective comparisons between transoral surgery and RT for oropharyngeal squamous cell carcinoma (OPSCC) confirm that the oncological results are similar, with surgical approaches leading to better patient-reported outcomes on functional results, at least in cases not requiring postoperative RT [30–32].

A meta-analysis performed in 2015 suggests that TORS and TOLS are equivalently efficacious as RT for the treatment of early OPSCC in terms of oncological outcome [30]. In more advanced-stage OPSCC RT plays a pivotal role and in an attempt to optimize oncological results, chemotherapy (CT) or cetuximab must be administered concomitant to RT [33]. In the early stages of disease (T1–T2 N0) the 5-year overall survival following conventional RT or TORS/TOLS as single modality treatment varies between 70 and 80% [34]. In advanced tumor stages, due to local (T3–T4) or regional extension (N1-3) the 5-year overall survival rates vary between 20 and 50%. Malignant salivary gland tumors of any histopathology (T1–T4a) are treated with surgery when possible. Postoperative RT is indicated for advanced-stage disease (T3–T4a), intermediate or high-grade tumors, close or irradical margins, lymph node metastases, bone invasion, perineural and vascular invasion, etc. [35]. Nasal and paranasal sinus carcinomas are usually treated with surgery followed by RT [36]. Due to the rarity and histopathological diversity of malignant paranasal tumors, there is no consensus on the therapeutic approach for these cancers [37].

HPV-related OPSCC plays a distinct role in HNC and has been recently recognized as a separate entity besides HPV-negative OPSCC resulting in HPV-specific staging concepts introduced in 2018 [7, 38]. This adjustment in staging was necessary because patients with HPV-associated OPSCC have a noticeably better prognosis than HPV-negative OPSCC [7, 39]. However, to date the HPV-status has not resulted in a de-escalation of treatment intensity. The 5-year overall survival rates for HPV-related OPSCC vary between 70% and above 80%, depending on disease stage [40]. Trials using cetuximab instead of cisplatin as de-escalation strategy for HPV-positive OPSCC showed inferior 2-year overall survival rates for cetuximab compared to cisplatin [7, 38]. In addition to the aforementioned treatment modalities, it is important to emphasize the need for a multidisciplinary setting of HNC care. A well-functioning, experienced MDT is essential for optimal planning, management, and delivery of care for HNC patients. The MDT typically comprises otolaryngologists/phoniatricians, plastic and reconstructive surgeons, maxillofacial surgeons, dental oncologists, radiation and medical oncologists, radiologists, SLPs, dietitians, physiotherapists, occupational therapists, and HNC nurse coordinator/clinical nurse specialists [41]. MDTs seek input from other disciplines including dental/maxillofacial prosthodontics/facial prosthetics, social work, pathology, physiotherapy, occupational therapy, psychology/psychiatry, palliative care, and counseling. Cancer care guidelines advocate that HNC services should be provided by the MDT housed in an established, patient-centered head-and-neck oncology center with a dedicated team of health professionals [42]. Protocols should consider the perspectives of MDT members for different tumor types and those in different professions [43]. Expertise in HNC is important with recommended clinical criteria and minimum patient volumes ensuring high standard care amongst different areas of expertise [44, 45]. A MDT approach results in improved patient outcomes and better survival rates [46–49], with improved care coordination, reduced time to treatment, and adherence to clinical guidelines [50]. It is recognized that delivery of MDT services through a coordinated head and neck clinical pathway (HNCP) maximizes results, increases efficiency in care delivery, reduces costs, shortens the length of hospital stay, and improves overall patient outcomes [51–55]. Coordination of a HNC MDT may be challenging [54], yet, despite its complexity, we argue that positive clinical, patient, and service outcomes can best be achieved through the implementation of a well-designed HNCP and MDT approach.

## Oropharyngeal dysphagia in head and neck cancer

### Pathophysiology and mechanisms of oropharyngeal dysphagia

Many HNC patients experience some degree of OD [56, 57]. The nature and degree of OD depend on the site and size of the primary tumor, with most severe complaints reported by patients with advanced oropharyngeal and hypopharyngeal lesions and those with cervical esophageal cancers [58]. Swallowing is a complex sensory-motor function. All stages of the physiologic swallowing process including motility, sensitivity, and biomechanical events may be altered in HNC patients. The oral preparatory phase (adequate lip closure and buccal tone for bolus containment, mastication and bolus formation), the oral phase (voluntary tongue strength, competent tongue function to move the bolus within the mouth and posteriorly), the pharyngeal phase (very rapid series of reflexive actions where the timing of biomechanical events is crucial), laryngeal elevation and anterior hyolaryngeal excursion with upper esophageal sphincter (UES) relaxation and bolus weight to open the UES, and the esophageal phase (completely involuntary) may be compromised. Changes in the lingual driving force, the pharyngeal clearing forces, the hypopharyngeal suction pump, and laryngeal competence may affect pharyngeal swallowing [59]. Pre-existing swallowing problems are usually worsened by the oncological treatment. Surgery may comprise resection of important swallowing structures, involving muscles and nerves leading to sensory-motor dysfunction and incoordination of swallowing, whereas (C)RT may lead to other pathophysiological changes. Damage to the oral cavity may lead to predictable but complex swallowing problems, such as difficulty with mastication, controlling food or liquid in the mouth, and initiating the pharyngeal swallow. Resections of up to one-third of the mobile tongue result in transient swallowing problems, but more severe problems occur when the tongue is tethered to the floor-of-mouth by the method of surgical closure, or when flap reconstruction leads to a non-innervated mass, modifying the spatial pattern and stereognosis of the oral cavity, or if sacrifice of the hypoglossal nerve is necessary [60]. Excision of the oropharynx causes more severe OD since the tongue base plays a critical role in initiating swallowing, propelling the bolus through the pharynx in combination with efficient pharyngeal peristalsis [61]. Any HNC treatment procedure that impacts oral containment of the bolus can result in premature loss of the bolus into the pharynx and aspiration risk [62]. This can subsequently result in delayed respiratory tract protection with aspiration before the swallow, reduced pressure generation, postswallow pharyngeal pooling, or reduced laryngeal elevation causing residue and postswallow aspiration especially in the presence of sensory deficits [63]. Combined resections of the soft palate and tonsillar pillars may affect pharyngeal bolus transport causing nasopharyngeal reflux and pharyngeal residue. Partial laryngectomy may be responsible for delayed and limited elevation of the larynx [64]. When (C)RT is employed frequent acute side effects include mucositis, edema, OD, and xerostomia [65, 66]. Posttreatment side effects decline within a few months in the majority of the patients. Late morbidity (> 6 months) is usually related to (C)RT and local damage caused by the tumor. Various degrees of fibrosis occur in the oropharyngeal muscles involved in the first two phases of swallowing. Sensory nerve function is often affected following HNC treatment thus modifying bolus propulsion, bolus localization, and oral stereognosis. Fibrosis of the pharyngeal constrictor muscles can lead to swallowing difficulties, symptomatic or silent aspiration and thus, often affects eating with considerable risks leading to feeding tube dependency [67–69]. As the salivary glands are often involved in the RT fields for oropharyngeal cancer, various degrees of xerostomia further complicate swallowing [70]. Being able to swallow following HNC treatment is one of the top functional priorities in patients and a driver for health-related quality of life (HRQOL) [71]. Modern RT techniques such as intensity-modulated radiotherapy (IMRT) allow for partial sparing of the parotid glands and the oral cavity and have the potential to lower the dose to the pharyngeal constrictors [72, 73]. The aim after oncological treatment is to return to oral feeding often with the help of an allied health professional and early speech therapy intervention to optimize swallowing outcomes [74]. Within the HNC population, the group of TLE patients represents a special sub-population. As the risk of choking is ameliorated in the absence of a larynx, these patients often receive less attention both in the clinic and in scientific OD research. In  "Oropharyngeal dysphagia following total laryngectomy" section the diagnosis and treatment of OD after a TLE is further described.

### Tracheostomy and oropharyngeal dysphagia in head and neck cancer

The need for a tracheostomy can be caused by the HNC itself or by the oncological treatment modalities such as (C)RT and surgery or combinations thereof. The upper respiratory tract can be compromised due to tumor obstruction, tumor destruction or paralysis of cranial nerves. HNC treatment can lead to lymphedema, fibrosis, and adhesion or synechia formation of the laryngeal mucosal membrane, which can compromise ventilation. Treatment often causes changes to saliva viscosity, which impairs the spontaneous handling of secretions in the throat leading to hypopharyngeal residue and salivary spilling into the larynx with subsequent aspiration.

Tracheostomy changes the anatomy and physiology of breathing, altering the timing and interaction of respiration and swallowing. This altered airway mechanism compromises the cough reflex and the ability to clear the airways due to changes in airflow and to the sensitivity of the larynx, which may affect the patients’ handling of secretions [75, 76]. Although a tracheotomy is usually performed to ensure breathing and ventilation, it is not without side effects. Besides the risk of bleeding and infection, which comprises both pneumonia or wound infection, other adverse events have been reported. These include secondary sinusitis, pneumothorax, subcutaneous emphysema, and persistent damage of the tracheal cartilage [77]. The one-year overall survival after tracheotomy in a mixed population in need for airway assistance (both neurological intensive care unit and HNC population) was 50% [78]. Assessment of OD in the presence of a tracheostomy tube remains subject of debate. The Modified Evan’s Blue Dye Test (MEBDT) is considered as a screening tool for aspiration in tracheotomized patients. However, the literature in this area is divergent and the MEBDT is not considered diagnostically accurate in the decision-making on OD management and/or the weaning procedure in HNC patients [79, 80].

Eating with a tracheostomy tube in situ has been evaluated in function of tracheostomy tube status (occlusion versus open tube). Leder and colleagues did not find significant differences in aspiration rate between these tube status conditions [81, 82]. Cuffed tracheostomy tubes are used to seal the airway when there is a need for positive pressure ventilation—or to prevent food or saliva entering the lower respiratory tract. Tracheostomy tubes or cannulas with a cuff might help in decreasing aspiration of secretions in the lower respiratory tract and can be used for tracheobronchial suction cleaning in case of severe aspiration. The evidence of whether a cuffed tube prevents further bolus aspiration into the lower respiratory tract while eating and drinking is still inconclusive [83]. Unfortunately, the clinical perception still persists that tracheotomy and placement of a tracheostomy tube increases the incidence of aspiration and decannulation causes improvement of swallowing function.

According to some studies, the tracheal tube cuff status (inflated versus deflated) might affect swallowing physiology too [84–86]. In a retrospective study of tracheotomized patients with mixed etiologies, subgroup analysis of the 102 HNC patients showed that postswallow residue and silent aspiration were the most frequent signs of swallowing impairment [84]. The authors found reduced laryngeal elevation and reduced laryngeal and pharyngeal sensitivity during cuff inflation. However, in a further study with tracheotomized patients, the authors did not find tethering of the larynx during videofluoroscopic swallow study (VFSS) using tubes with and without cuff [86]. This finding was confirmed later by Leder and colleagues where pre- and post-tracheotomy swallowing evaluation failed to show a direct causal relationship between tracheostomy and aspiration status [87]. In conclusion, the presence of OD is usually caused by the underlying disease or condition rather than by the tracheostomy itself. It is suggested here that this is also the case for HNC patients who have undergone extensive HNC treatment for advanced HNC stages irreversibly affecting their upper aerodigestive tract. Most experts agree that in the context of best clinical practice a MDT should assess secretion handling, swallowing, and the weaning procedure in tracheotomized HNC patients using fiberoptic endoscopic evaluation of swallowing (FEES) and/or VFSS (see "Videofluoroscopic swallow study " and "Fiberoptic endoscopic evaluation of swallowing" sections) [88].

## Screening and clinical assessment of oropharyngeal dysphagia in head and neck cancer

The first step in the management of patients at risk for OD is screening. The purpose of screening is to identify patients at risk for aspiration or swallowing problems. Any HNC patient at risk of OD should be screened. HNC patients at risk of OD are usually screened on their initial pretreatment appointment before the onset of oncological treatment. Screening results in a pass or a fail; patients are deemed to be either at risk (screen failed) or are not at risk (screen passed). Patients who fail screening must be referred for further assessment to evaluate the swallow physiology and functioning and, if possible, determine the cause of swallowing problems [89]. Depending on the acuteness and stability of a patient’s health, screening must be repeated to confirm or adjust previous screening outcomes. Patients who passed earlier screening, but are subject to changing health conditions due to for example aging or the effects of oncological treatments may be at risk of developing OD over time. Screening measures must be valid, reliable, and feasible to administer. Recent diagnostic reviews provide overviews of existing screening measures and their diagnostic performance (e.g., sensitivity, specificity, positive predictive value, negative predictive value, and likelihood ratios). Several reviews recommend water swallowing tests using different endpoints (e.g., choking, coughing) [90–95] to determine whether patients passed or failed screening. Even though these reviews did not specifically target HNC patients, the use of a water swallowing test seems to be the most appropriate choice in the absence of diagnostic reviews involving HNC patients only.

Given that OD is a multi-faceted phenomenon [96, 97], different dimensions of swallowing do not necessarily correlate. As such, a multidimensional approach to the assessment of OD is needed, including medical and patient history taking; conducting instrumental assessments including FEES or VFSS, and administering clinical assessments and patient self-reports [89]. Medical and patient history information will provide information on factors associated with OD, including (but not limited to): respiratory impairment and use of medication; the presentation (or possibly representation) of pneumonia and sudden unexplained weight loss, etc. Clinical assessment comprises a broad range of assessments, including the functional assessment of cognition and communication abilities; the evaluation of the oral, laryngeal, and pharyngeal functioning in terms of physiology, anatomy, and neurology, with a specific focus on cranial nerve function; oral intake; and oral health. The dietary intake and nutritional status can be reviewed by assessments such as the Functional Oral Intake Scale (FOIS) [98] or the Mini Nutritional Assessment (MNA) [99]. Mealtime observations and trial swallows, using different bolus consistencies and volumes, possibly in combination with postural adjustments and swallowing maneuvers, are conducted to evaluate safety and efficacy of swallowing and options for OD treatment [89, 100]. However, instrumental assessment is required, for example, to rule out silent aspiration or retrieve more detailed information about the swallowing act.

Patient self-evaluation of functional health status (FHS) and HRQOL are conducted by means of self-administered questionnaires. FHS refers to the influence of a given disease or OD on particular functional aspects – for example – an individual's ability to perform normal daily activities such as eating required to meet basic needs, fulfill usual roles, and to maintain health, and well-being [101]. HRQOL, conversely, is the unique personal perception of someone’s health, taking into account social, functional, and psychological aspects [102]. Many questionnaires, however, combine FHS and HRQOL without making a distinction between the two constructs. Commonly used HRQOL questionnaires in oral health (e.g., 5-item Oral Health Impact Profile or OHIP-5) [103] are not specifically developed for patients with HNC, with the exception of the oral health supplementary questionnaire module by the European Organization for Research and Treatment of Cancer Quality (EORTC QOL-OH15) [104]. It is recommended that the EORTC QOL OH-15, which focuses on oral health, should be administered in conjunction with the HRQOL questionnaire EORTC QLQ-C30. This EORTC QLQ-C30 focuses on general quality of life (QOL) in HNC patients [105]. Still, very few questionnaires specifically target OD-related QOL in HNC patients. Examples include the MD Anderson Dysphagia Inventory (MDADI) [106] and the QLQ-H&N35, which has a 4-item subscale on swallowing [107].

Table 1 provides examples of commonly used screening and clinical assessments in HNC patients with OD. However, when selecting measures, only those with robust psychometric properties should be selected, otherwise patient data cannot be interpreted as valid and reliable and no clinical inferences should be made [108, 109]. Recent psychometric reviews on measures in OD have indicated their psychometric status as either poor or lacking data on validity, reliability, and responsiveness in, for example, measures on FHS, HRQOL or visuoperceptual evaluation of FEES and VFSS [110–112]. Item Response Theory (IRT) has been introduced as a contemporary methodology to interrogate the psychometric quality of measures and should be used in combination with Classical Test Theory (CTT) [109, 113, 114]. Although procedures and interpretation of CTT are relatively straight forward compared with IRT, CTT has some limitations. The CTT framework evaluates the performance of the measure as a whole and is specific to the sample population the measure was tested with. By contrast, in IRT the unit of analysis is the item and results are not bound by the test population [114, 115]. In recent years, IRT has been used to evaluate and critique measures commonly used in OD which calls for the development of new measures or the refinement of existing measures [116–119]. Future research should focus on comprehensively evaluating all psychometric properties for existing measures and developing new measures validated in patients with HNC using contemporary standards for instrument development, such as COnsensus-based Standards for the selection of health Measurement INstruments (COSMIN) [120].

**Table 1:** Examples of commonly used screening and clinical assessments in head and neck cancer patients with oropharyngeal dysphagia

Domain	Screening and clinical assessment^a^	Acronym	References
At risk of swallowing problems	Screening: water swallowing test using different endpoints, e.g.:		
	100 ml Water Swallow Test^b^	100 ml WST	[460]
	Toronto Bedside Swallowing Screening Test	TOR-BSST	[461]
	Volume-Viscosity Swallowing Test	V-VST	[462]
Cognition and Communication	Mini-Cog Test		[463]
	Mini-Mental State Examination	MMSE	[464]
Nutritional status	Malnutrition Universal Screening Tool	MUST	[465]
	Mini Nutritional Assessment Simplified Nutritional Appetite Questionnaire	MNA SNAQ	[99] [466]
Oral intake status	Functional Oral Intake Scale	FOIS	[98]
Health-related quality of life	Quality of Life Questionnaire (by the European Organization for Research and Treatment of Cancer Quality)^b^	EORTC QLQ-C30	[105]
Dysphagia-related quality of life	MD Anderson Dysphagia Inventory^b^	MDADI	[106]
(including functional health status)	Symptom scale Swallowing of the Head and Neck Cancer Module (by the European Organization for Research and Treatment of Cancer Quality)^b^	Symptom scale Swallowing of the EORTC QLQ-H&N35	[107]
Dysphonia-related quality of life (including functional health status)	Voice Handicap Index	VHI	[467]
Oral health status	5-item Oral Health Impact Profile	OHIP-5	[103]
	Oral Health supplementary questionnaire module (by the European Organization for Research and Treatment of Cancer Quality)^2^	EORTC QOL-OH15	[104]
Anatomy/cranial nerve integrity	Clinical examination of the tongue, hard and soft palate, teeth, gums, oral mucosa, trigeminal (V), facial (VII), glossopharyngeal (IX), vagal (X), and hypoglossal (XII) cranial nerves		
Oral motor skills/physiology	Clinical examination of oral muscle strength, range, tone, steadiness, accuracy, and coordination		
	Mealtime observation including observation of drooling or sialorrhea, mastication, eating speed, cough or choking, oral residue, head and body positioning		
Compensatory strategies/maneuvers	E.g.: bolus modification, postural adjustments and/or swallow maneuvers		

^a^No international consensus exists on which screening or assessment for dysphagia is preferred in head and neck cancer patients [97]. In addition, many screenings and assessments have unknown or poor psychometric properties or have not been validated for head and neck cancer patients. The presented list of screenings and assessments does not provide a complete overview, but examples of common clinical practice

^b^Targeting patients with head and neck cancer

## Instrumental swallowing assessment in head and neck cancer

### Videofluoroscopic swallow study

As the findings from both feeding status of the patient and patient-reported outcomes may not always be associated with findings from VFSS assessment in HNC patients [121–123], instrumental swallow evaluation is critical to provide prompt OD management. VFSS is a dynamic radiological imaging examination, which provides direct visualization of bolus flow and structural movement during the oral preparatory, oral, pharyngeal, and upper esophageal stages of swallowing. VFSS is employed to evaluate oropharyngeal swallowing and to guide interdisciplinary OD intervention. VFSS can capture the impact of HNC, surgical intervention, and (C)RT on oropharyngeal swallowing over time. The esophageal phase of swallowing can also be screened during VFSS to determine the need for onward referral and more in-depth esophageal evaluation [124]. VFSS should be selected over FEES when oral or esophageal phase dysphagia is suspected based on the clinical evaluation. During the VFSS protocol, lateral and anterior–posterior views are obtained. Initially, the patient is seated in the lateral position and an optimum view should be obtained to evaluate oropharyngeal swallow anatomy at rest to rule out any structural abnormalities or complications resulting from HNC or surgical treatment (e.g., tracheo-esophageal fistula). Standardized liquid bolus volumes impregnated with barium should be administered with the patient in a head neutral position. Method of bolus delivery can alter swallowing and patients should be encouraged to self-feed from a cup or spoon where possible to replicate real-life eating behavior. When safe, larger liquid bolus volumes can rule out cricopharyngeal and upper esophageal pathologies (e.g., posterior cricoid web, cricopharyngeal bar, laryngo-pharyngeal reflux) and can expose impaired UES opening during swallowing due to post-RT fibrosis of suprahyoid, pharyngeal constrictor or cricopharyngeal musculature [124–126]. Where safe, a range of bolus consistencies (thin liquids, nectar thick, pudding, solid) should be trialed during VFSS. Solid foods may be challenging for patients with impaired mastication resulting from trismus; weak lingual range of movement secondary to RT-induced fibrosis; xerostomia or altered taste. An anterior–posterior view should also be included during the VFSS protocol to evaluate the symmetry of pharyngeal or upper esophageal residue and to observe vocal fold adduction. Finally, an esophageal screen is critical during VFSS. Despite the fact that VFSS is not very sensitive to detect esophageal cancer and gastro-esophageal reflux disease, it can still contribute to a global assessment of the esophageal phase of swallowing and the need to refer the HNC patient to the gastroenterologist [127]. Of note, VFSS pulse rate should be considered as VFSS findings differ using a rate of 30 pulses per second compared to 15 pulses per second [128, 129].

Where aspiration or residue is observed during VFSS, compensatory strategies should be trialed before introducing modified diet consistencies. While the evidence base for compensatory strategies is very limited in the HNC population [130], postures (e.g., chin tuck, head turn) and maneuvers (e.g., effortful swallow, supraglottic swallow, Mendelsohn maneuver) can alter swallow safety and efficiency and are often preferred by people with swallowing difficulties to diet modification [131]. As many patients with OD secondary to HNC already present with weight loss, decreased appetite, taste changes, and malnutrition, these strategies should be tested during VFSS.

In terms of VFSS interpretation, validated rating scales should be employed to monitor swallowing performance over time and to establish any response to intervention (see "Screening and clinical assessment of oropharyngeal dysphagia in head and neck cancer" section). Swallow safety can be rated using the Penetration Aspiration Scale (PAS) [132]. This is a visuoperceptual eight-point ordinal rating sale, which measures the depth of airway invasion and the presence and efficiency of a cough response to aspiration [132]. Swallow efficiency is commonly rated using validated perceptual or quantitative residue scales including the Bolus Residue Scale [133] and the Normalised Residue Rating Scale [134]. The Dynamic Imaging Grade of Swallowing Toxicity (DIGEST) is a five-point rating scale recently developed and validated for the HNC population, which captures both swallow safety and efficiency [135]. To provide safe and beneficial OD rehabilitation, clinicians must look beyond aspiration and residue during VFSS interpretation to identify the underlying pathophysiology. Tools such as the Modified Barium Swallow Impairment Profile (MBSImp), a standardized VFSS scoring system, which involves rating various physiologic components of swallowing [136], can help to identify the underlying physiological cause of OD. Components typically altered in HNC patients include anterior and superior hyoid excursion, UES opening, initiation of swallowing, tongue base retraction, and pharyngeal residue [121]. As described in "Screening and clinical assessment of oropharyngeal dysphagia in head and neck cancer" section there is still room for improvement of the psychometric properties of VFSS measures in patients with HNC.

### Fiberoptic endoscopic evaluation of swallowing

FEES, first described by Langmore in 1988, allows the anatomical assessment of the pharynx and larynx, providing an excellent visualization of the anatomical and physiological changes after HNC surgery and/or (C)RT. Inspection of the UES is also possible but it requires frame by frame analysis and is easier in cases of patent UES. FEES also allows a comprehensive evaluation of the pharyngeal stage of swallowing [137, 138]. Together with the VFSS, FEES is the most commonly recommended method of instrumental swallowing assessment for HNC patients in many guidelines [139–141]. It is a safe procedure with a reported low incidence of complications in patients with OD of mixed etiology [142].

To perform a FEES examination both clinical skills in handling the endoscope and knowledge on swallowing anatomy, (patho)physiology, and rehabilitation are required [143, 144].

Currently, there is no consensus in the literature on a FEES protocol for HNC. However, studies on this topic [145–147] recommended a comprehensive evaluation of swallowing that includes the assessment of the anatomy of the upper aerodigestive tract, sensibility of the larynx, motor control of swallowing structures, secretion management, effect of bolus modification (volumes and consistencies) and swallowing maneuvers. The assessment of the upper aerodigestive tract; plays a critical role in HNC patients as it allows appraisal of surface mucosa as well as the structural integrity and changes in swallowing structures. Surface mucosa assessment is a medical act of pivotal importance as it may reveal diseases, such as mycosis or recurrent carcinoma requiring prompt diagnosis and treatment. Both surgical and non-surgical HNC treatment often lead to significant structural changes, such as UES stricture formation or patency, pharyngeal tissue thickening (edema and/or fibrosis), volume changes of the tongue base and pharynx, etc., that require anatomic and functional assessment. Sensibility of the larynx and motor control of the velopharyngeal sphincter, pharynx, tongue base, and larynx during non-swallowing tasks should be carefully assessed [148–150]. Sensory impairments in the larynx after (C)RT are common and may contribute to aspiration [151]. However, further research is needed to determine the late effects of HNC treatment, in terms of prevalence – biomechanism – etc., on the oropharyngeal and laryngeal sensory function.

Secretions and swallowing performance with different bolus volumes and consistencies are mandatory during a FEES examination. The presence of secretions in the larynx of HNC patients is a common finding and it can be associated with a high risk of aspiration [152]. There is no consensus on the number of swallow trials, bolus volume or consistencies applied during FEES. Studies reported that the number of swallow trials necessary to reveal aspiration for thin liquid is smaller in HNC patients when compared to neurology patients [153]. In both patient populations, the aspiration risk is underestimated when using a limited number (three or four) of swallow trials. At least 6 swallow trials with thin liquids and 4 swallow trials with thickened liquids should be assessed to avoid underestimation of aspiration probability [153]. Different bolus consistencies should be offered during FEES as swallowing performance changes according to the consistency [154]. Swallow trials of different bolus volumes and consistencies are crucial for decision-making on oral intake. However, to understand the swallowing pathophysiology all aspects involved in swallowing (anatomy, sensibility, motor control, secretion management, and swallowing performance) should be assessed.

The effect of different interventions (diet modification, application of compensatory strategies, and airway protective swallow maneuvers) on swallowing function should be assessed during FEES as it can guide decision-making on oral feeding and the rehabilitation program [138].

There are several advantages of using FEES in HNC patients; for instance, it allows the assessment of swallowing, voice, and local tumor recurrence in the same examination. Although FEES has been extensively applied in HNC patients [145], particularly after open partial laryngeal surgery [155], standardization and validation of FEES rating scales for HNC populations require further development (see "Screening and clinical assessment of oropharyngeal dysphagia in head and neck cancer" section) [111]. Given that HNC patients often present with anatomical and functional changes in the oropharyngeal structures, as well as a high prevalence of comorbidity, the psychometric properties of existing FEES scales cannot simply be extrapolated to a HNC patient population. FEES rating scales, such as the PAS [132, 156] and the Yale Pharyngeal Residue Rating Scale [157], were not specifically developed for HNC patients. An exception is the adaptation of the PAS for patients who underwent partial laryngectomy [158] and the scale developed to evaluate swallowing residue post-TLE [159]. Although the level of intra- and interobserver agreement of the FEES rating scales was reported to be similar in neurology and oncology patients [160] there is insufficient evidence that FEES scales developed and validated for neurogenic OD are also suitable to evaluate swallowing in HNC patients. Further studies should analyze the psychometric characteristics of existing FEES scales in HNC patients or develop and validate FEES protocols specifically for this population.

FEES and VFSS are both well-established instruments to assess swallowing and their advantages and disadvantages have already been described in the literature [161, 162]. The main differences are that only FEES is able to assess the surface mucosa, saliva residue, and laryngeal sensory function; it can be performed at the bedside and repeated as often as needed as there is no radiation exposure. On the other hand, only VFSS allows oral and esophageal phase visualization, including the assessment of oropharyngeal transit timing and UES opening. Findings of simultaneous FEES and VFSS examination have shown that FEES may lead to a more severe impression of pharyngeal residue based on a superior visualization of the bolus [163–165]. Studies on the correlation between FEES and VFSS in HNC patients have shown similar results regarding outcomes on pharyngeal residue [159, 166]. However, aspiration was perceived as less severe during FEES in HNC patients probably due to the restricted view of the larynx based on among others mucosal edema [166].

### Manometry

In the past, high-resolution manometrie (HRM) has been used to investigate upper gastrointestinal, usually esophageal function. In the last decade, HRM is increasingly applied to evaluate the muscular function of the pharynx and the UES during deglutition [167]. HRM measures contraction of the entire pharynx and the UES segment using a transnasal catheter with closely spaced pressure sensors. HRM can be combined with VFSS and/or impedance measurements and has been incorporated into commercially available diagnostic systems.

The pharyngeal phase of swallowing consists of a series of actions, which can be identified using a HRM color plot. Firstly, laryngeal elevation can be seen by an upward movement of the UES. Secondly, the pharyngeal contractile wave is registered as a peristaltic sequence of pressure increase over time along the entire pharynx. Thirdly, the UES relaxation is identified by a pressure drop overtime at the UES high-pressure zone with a recovery back to UES resting pressure baseline. Finally, the proximal esophageal contraction can be recognized by an increased pressure in the UES and the proximal striated part of the esophagus [168].

The UES compliance depends on both UES relaxation and UES opening, which are different yet closely interlinked. UES opening is an active neuromuscular event rather than a single consequence of UES relaxation and the diameter of the opening varies according to bolus volume and consistency [169–171]. It is important to recognize that HRM as a standalone technique allows measurement of UES relaxation, but is unable to measure UES opening in terms of the diameter.

When HRM is recorded simultaneously with intraluminal impedance measurement, combined manometry impedance patterns can be analyzed in an integrated fashion using pressure-flow analysis (PFA). PFA derives pharyngeal pressure-impedance variables, which are objective metrics of deglutitive function and are altered in relation to impaired swallowing [172, 173]. Aspiration risk can be assessed through a swallow risk index (SRI), which is a formula combining four pharyngeal pressure-impedance parameters relevant to aspiration [172, 173]. In neurogenic OD as well as in HNC patients, the reliability and validity of the PFA-derived SRI and the measure of postswallow residue were evaluated by comparing them against VFSS as the criterion reference standard. Studies have shown that PFA-based measures of swallowing function have good interobserver reliability and that these measures are easily determined and are objective markers of clinically relevant features of OD [174, 175]. Nowadays, the use of HRM for assessment of pharyngeal and UES motor function is common in clinical practice. Recently, a High-Resolution Pharyngeal Manometry International Working Group reached a consensus on methodology, protocol, and metrics for high-resolution pharyngeal manometry (HRPM) with consideration of impedance as an adjunct modality [176]. Normative values and online analysis methods are now available and a classification system for UES motility disorders has been developed [177]. HRM has significant potential in determining the pathophysiology of OD in HNC patients and future studies could potentially benefit highly from including these objective measurements.

## Complications due to oropharyngeal dysphagia in head and neck cancer

This section deals primarily with airway-related problems due to OD. Other consequences of OD are discussed in separate sections. The topic of positive short- and long-term outcomes for upper aerodigestive tract function associated with low complication rates is one of increasing importance [178, 179].

Both surgical and non-surgical treatment of HNC can result in transient or permanent OD as described in "Pathophysiology and mechanisms of oropharyngeal dysphagia" section [178]. OD puts the patient not only at risk for aspiration pneumonia, malnutrition, dehydration, and choking, which all increase morbidity but also for reduced QOL [180]. Incidence, diagnosis, and management of malnutrition and dehydration are described in further detail in "Cancer cachexia in head and neck cancer" and "Treatment of cancer cachexia" sections. Aspiration pneumonia is recognized as pneumonia secondary to the entrance of food particles, saliva, or gastric acid into the lower respiratory tract [181]. Definitions for aspiration pneumonia vary according to the literature. Kawai and colleagues defined aspiration pneumonia as the presence of wet cough, sputum, and fever in addition to coarse crackles in the chest, elevated inflammatory serum markers and image findings (X-ray or computed tomography scan of the chest) [181]. The incidence of aspiration pneumonia within a year after (C)RT differs, ranging from 5.4 to 23% [182–184]. Variation in incidence may be due to a higher risk of aspiration pneumonia following CRT or cetuximab with concurrent RT than following RT as a single modality treatment [185]. Malnutrition with hypoalbuminemia is described as a predictive factor for aspiration pneumonia after (C)RT as hypoalbuminemia negatively affects the immune system [185]. Further predictive factors for aspiration pneumonia following (C)RT are poor oral hygiene (OH), use of sleeping pills, coexistence of other malignancies, and habitual alcohol consumption [181]. Szczesniak and colleagues reported that approximately 52% of the patients who received RT and 69% who received (C)RT suffered from long-term OD after treatment, and aspiration pneumonia accounted for 19% of non-cancer-related deaths [186]. Therefore, clinicians should assess the risk of aspiration pneumonia to identify patients for whom efforts to prevent aspiration pneumonia should be intensified [181]. Screening for aspiration with a clinical swallowing test for HNC [e.g. Mann Assessment of Swallowing Ability-Cancer (MASA-C)] and subsequently verifying positive screening results with a FEES or VFSS assessment, should be performed if aspiration is suspected ("Screening and clinical assessment of oropharyngeal dysphagia in head and neck cancer", "Videofluoroscopic swallow study", and "Fiberoptic endoscopic evaluation of swallowing " sections) [187–189]. The body of literature on comparative incidence and duration of aspiration following conventional HNC surgery, TOLS or TORS remains limited at present [178]. This is the subject of ongoing clinical trials. Aspiration should be anticipated and avoided as far as possible by using airway protective swallow maneuvers under the guidance of specialized SLPs, general patient education, bolus modification ("Bolus consistency modification" section), and sometimes tracheostomy for bronchopulmonary suction ("Tracheostomy and oropharyngeal dysphagia in head and neck cancer" and "Surgical treatment of oropharyngeal dysphagia in head and neck cancer" sections) [151, 181].

## Cancer cachexia in head and neck cancer

Cancer cachexia is defined by Fearon and colleagues is *“A multifactorial syndrome characterized by an ongoing loss of skeletal muscle mass (with or without loss of fat mass) that cannot be fully reversed by conventional nutritional support and leads to progressive functional impairment”* p. 490 [190]. The pathophysiology is characterized by disturbances in the whole body and cellular energy metabolism, muscle protein turnover regulation, and impaired muscle regeneration [190, 191]. Next to these metabolic disturbances patients often suffer from a reduced oral food intake and appetite [190, 192]. Within the HNC population, the incidence of cachexia varies from 6.1 to 66%, depending on the time in relation to treatment and cachexia is more pronounced in locally advanced tumors [193–195]. Multiple studies have shown that cachexia is related to decreased therapy tolerance, higher treatment toxicity, and above all worse overall survival and progression-free survival [193–197]. OD is one of the factors leading to a reduced oral food intake in HNC patients [198]. Furthermore, catabolic processes causing muscle atrophy [199] might not only affect peripheral skeletal muscles but also swallowing muscles. The muscle wasting component in HNC patients’ OD is still underexplored, but OD due to sarcopenia (sarcopenic OD) in the elderly population has been reported [200–203]. Additionally, in cancer patients, an association has been found between skeletal muscle mass and severe OD [204, 205]. Feng and colleagues showed that the cross-sectional area of the geniohyoid muscle, as surrogate for swallowing muscle volume, was significantly lower in aspirating elderly men compared to non-aspirators [206]. These studies support the hypothesis that metabolic derangements in cancer cachexia not only affect overall skeletal muscle mass but also affect swallowing muscle mass in particular, leading to OD. Recently, diagnostic criteria for sarcopenic OD were proposed, but remain difficult to apply in HNC patients [207]. The authors described that a definite diagnosis of sarcopenic OD can only be obtained if imaging is consistent with a loss of swallowing muscle mass [207]. Unfortunately, imaging of swallowing muscles remains challenging. Only a few studies have managed to determine swallowing muscle volume through magnetic resonance imaging (MRI), computed tomography scan, and ultrasound [208–210]. Swallowing muscle function can be assessed more readily through, among others, tongue-pressure tests [211]. Besides evaluating swallowing muscle function, Maeda and colleagues found other potential clinical predictors for the development of sarcopenic OD. The combination of performance status, ambulatory status, nutritional status, and low amount of oral intake can be used to screen patients at risk of developing sarcopenic OD following hospital admission [212]. Since the presence of cancer cachexia greatly influences treatment toxicity and patient-reported outcomes, screening for muscle wasting in HNC patients is recommended [213–215]. Body mass index (BMI) measurement cannot reveal a low muscle mass, in particular not when hidden by relative or absolute fat mass abundance. Ideally, a rapid screening method for body composition, such as bioelectrical impedance analysis (BIA) would be suitable for this purpose. It has been shown that measurements of the fat-free mass index using BIA have a prognostic value [198]. In addition, dual-energy X-ray absorptiometry (DEXA) is minimally invasive and enables adequate information on body composition. For study purposes, the use of cross-sectional muscle area on computed tomography scans provides insight in skeletal muscle mass and quality [216]. Future studies are needed to further establish the relationship between skeletal muscle wasting and swallowing muscle wasting in the HNC population. The treatment of cancer cachexia is considered further in "Treatment of cancer cachexia" section.

## Psychosocial and economic impact of oropharyngeal dysphagia in head and neck cancer

### Psychosocial impact

Psychosocial distress is defined as “*a multifactorial unpleasant experience of a psychological (ie, cognitive, behavioral, emotional), social, spiritual, and/or physical nature that may interfere with the ability to cope effectively with cancer, its physical symptoms, and its treatment*”[217]. Psychosocial disruptions can have a significant impact on HRQOL [218], which reflects the patient’s perception of cancer impact on a broad range of well-being domains, including physical, social, and psychological [102]. HNC has been identified as the most psychologically traumatic cancer for patients and their caregivers and is listed as one of the cancers associated with high risk for depression [217, 219, 220]. In addition to common stressors related to cancer diagnosis and its associated anxiety, pain, fatigue, and fear of recurrence, HNC patients can experience profound changes in physical appearance, speech, voice, swallowing, hearing, breathing, and high levels of symptomatology. These sequelae have detrimental effects on patients’ emotional well-being, self-identity and interpersonal relationships, and have profound implications for their QOL [221]. Compared to other oncological populations, it is suggested that HNC patients experience higher levels of depression and anxiety [222], heightened sense of uncertainty and hopelessness [223], and are at higher risk of suicide [224]. Facial disfigurement and the stigma related to it represent principal sources of distress, insecurity and shame, progressively leading to social withdrawal [225, 226]. Eating difficulties are often associated with emotional and social losses for HNC patients who report feelings of embarrassment, frustration, anger, and anxiety related to food and mealtimes [227–229]. OD has also a significant effect on caregivers, who need to adapt their own diets and can experience high levels of distress related to mealtime preparation and feeding tube management [230]. Changes in voice quality and speech intelligibility affect patients’ willingness to engage in communicative interactions and social activities [231, 232]. Evidence suggests that such changes have further negative impacts on employment opportunities, ability to work and return to the workplace (see "Reimbursement of head and neck cancer related oropharyngeal dysphagia costs in European countries" section) [233, 234]. All these and further psychosocial stressors, like secondary head and neck lymphedema [235], shoulder dysfunction [236], ongoing concerns of cancer recurrence [237], sexual dysfunctions [238], the high financial impact of the disease [239], ultimately contribute to poor HRQOL outcomes for this patient population and their family and caregivers.

Psychosocial interventions have been demonstrated effective in improving HRQOL in different cancer populations [240, 241] and psychosocial support is recommended by international clinical guidelines for cancer care [45, 242, 243]. Specifically for the HNC population, psycho-educational programs, cognitive behavioral training, psychotherapy, group support, and pharmacological interventions for both patients and caregivers have been proposed [218, 244, 245]. While the evidence supporting the effectiveness of such interventions is lacking [246], counseling and patient education, peer and social support are perceived as critical factors in alleviating psychological distress and increasing confidence. Referral to psychosocial health professionals, including psychologists, social workers, counselors, nurses, and psychiatrists, is recommended before, during and/or after cancer treatment and is advocated by national guidelines and policies for HNC [15, 247]. The role of online support groups is also recognized (see "Head and neck cancer patient perspective" section) [248]. Because HNC patients often underreport their concerns and suffering they may be more reluctant to accept psychosocial support [249]. Screening and ongoing monitoring for psychosocial distress are critical. Screening measures such as the Distress Thermometer [217], the Hospital Anxiety and Depression Scale [250], or the Brief Symptom Inventory-18 [251] are most widely used in cancer populations. HRQOL measurements give a fundamental perspective on cancer treatment outcomes, by assessing the patient’s well-being over cancer trajectory [252]. Several validated questionnaires are routinely implemented in clinical practice to evaluate HRQOL in the HNC population [253]. These include general multidomain questionnaires, like the EORTC QLQ-H&N35 [107], the FACT-H&N [254], and the MDASI-HN [255], and questionnaires addressing specific HNC-related symptoms, for example, the MDADI [106] as described in "Screening and clinical assessment of oropharyngeal dysphagia in head and neck cancer" section. Addressing the psychosocial sequelae of HNC is paramount to improve patients’, carers’, and families’ QOL. It is recommended to incorporate psychosocial interventions early into HNC care pathways with screening procedures implemented into clinical routine for early identification and management.

### Reimbursement of head and neck cancer related oropharyngeal dysphagia costs in European countries

Acute in-hospital and long-term healthcare costs arise for HNC patients who have OD with their associated nutritional and respiratory complications. Charges also include direct non-healthcare expenditure, such as social costs, and indirect costs related to productivity loss, and intangible costs related to loss of income. A recent systematic review estimates the cost of OD as approximately €13.000 (approx. USD 14,900) in the acute phase of the disease and the expenditure related to an episode of aspiration pneumonia to be up to € 26.000 (approx. USD 29,800) in patients with post-stroke OD [256]. However, no systematic reviews are available on costs related to OD associated with HNC and its main complications, with very few studies published in the area. Short-term outcomes and costs of care after HNC surgery in the presence of OD is associated with a significant increase in costs during hospitalization with an increase of 2 days in the length of hospital admission for OD-related problems [257, 258]. An additional study examined the association between quality of care, short- and long-term outcomes related to the HNC treatment, and costs in elderly patients (66 years and older; *n* = 2370) treated for laryngeal cancer. Costs included in-hospital, outpatient, and long-term healthcare costs during a 5-year follow-up period. They found lower OD-related healthcare costs in those patients who received a higher quality standard of care using evidence-based practices associated with quality indicators [259]. In addition, a 2014 study reported an increase in OD related expenditure of USD 65,766 attributed to in-hospital, outpatient, physician/supplier, hospice, home healthcare, and medical equipment costs incurred over a 5-year follow-up period [260]. In summary, there is emerging evidence on the strong impact of OD on health-related economic costs. There is an urgent need for specific studies to further define the independent effect of OD on the healthcare costs of patients with HNC.

## Oropharyngeal dysphagia treatment in head and neck cancer

### Bolus consistency modification

Bolus consistency modification is a compensatory strategy for HNC patients with OD following oncological treatment that has two main aims: (a) to maintain a safe way of oral hydration (by fluid thickening); and (b) to maintain patients’ nutritional status with texture modified foods. The prevalence of malnutrition is high in HNC patients with OD as described in "Cancer cachexia in head and neck cancer" section [56]. Bolus consistency modification should be adapted to the mastication and swallowing ability of HNC patients, and include: (a) rheological adaptation; for fluids—viscosity and for solids—texture adaptation for safe swallowing, (b) nutritional adaptation to meet the nutritional requirements of HNC patients with OD, and (c) organoleptic adaptation to optimize taste, smell, appearance, and palatability of the food and fluids. These considerations should be applied systematically during the treatment of OD in HNC patients to improve their nutritional status and HRQOL [261]. But these considerations should also be applied to HNC patients who are still on the inpatient ward after, for example, a surgical intervention whereby stepwise reintroduction of oral food intake is often applied. In the majority of HNC patients, the modification of bolus consistency of solids and/or liquids will be applied in conjunction with rehabilitative biomechanical OD interventions (see "Head and neck cancer specific exercise treatment for oropharyngeal dysphagia" section) [262].

#### Fluid thickening (liquids)

**Fig. 1:**
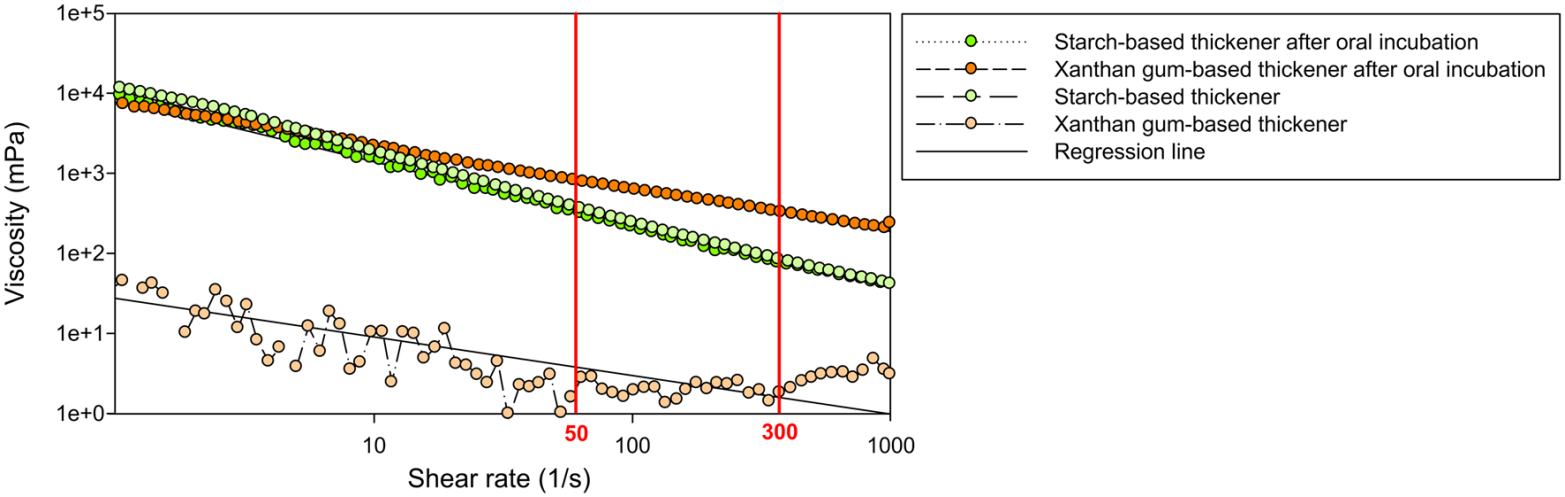
Shear thinning and amylase resistance of a xanthan gum-based (XGB) and a starch-based (SB) thickening agent. Note the shear thinning between 50 s^−1^ and 300 s^−1^ is quite comparable between XGB and SB agents; however, the reduction of viscosity caused by 30 s incubation with salivary amylase is much higher for the SB thickening agent

Fluid bolus is characterized by its basic rheological property namely viscosity, which defines its resistance to flow. The effect of salivary amylase in the oral phase and shear thinning (defined as the non-Newtonian behavior of fluids whose viscosity decreases under shear strain) during the pharyngeal phase of swallowing are the two main factors affecting the safety and therapeutic effect of thickened fluids. Starch-based (SB) and xanthan gum-based (XGB) thickeners are often used in diet modification for HNC patients with OD. However, the hydrolysis of SB components by α-amylase reduces the viscosity of the thickened drinks prior to swallowing resulting in a negative treatment effect of OD (Fig. 1) [100, 263]. A White Paper from the ESSD concluded that there is evidence to suggest that increasing bolus viscosity can lower the risk of aspiration and that it is a valid management strategy for OD [100]. Bolus consistency modification in HNC patients with OD can improve several pathophysiological consequences without improving the actual swallowing function. This can manifest, for example, as improving: (i) the bolus formation [264], (ii) impaired oropharyngeal bolus propulsion following treatment for oropharyngeal cancer at subsites such as the tongue base, pharyngeal wall, and soft palate or (iii) aspiration due to impaired upper respiratory tract closure and sensorial feedback in case of laryngeal dysfunction following (C)RT or partial laryngectomy. Nonetheless, there is a need for research to establish the ‘optimal’ viscosity levels to prevent aspiration and improve the safety and efficiency of oral intake for HNC patients [154, 265]. Thickened fluids and altered consistency foods can also impact negatively on HRQOL (see "Psychosocial impact" section).

**Fig. 2:**
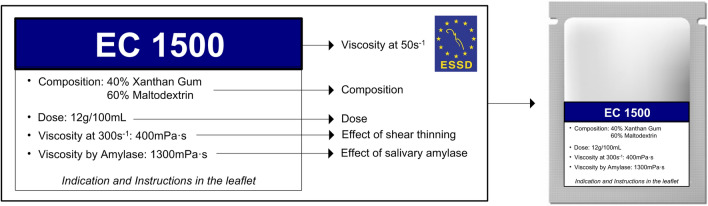
The ESSD proposes to go further than just the use of a descriptive classification of viscosity levels and therefore recommends that manufacturers of thickening agents also include the viscosity values in Système Internationale d’Unités (SI) units (mPa s) into the labels of thickening agents as presented by this example

Previous viscosity classification models using descriptive textural names (‘nectar’, ‘honey’ or ‘spoon thick’) show a strong disagreement between health professionals regarding the interpretation of these qualitative descriptors [266]. Different classifications of viscosity levels have emerged in recent years such as the National Dysphagia Diet (NDD) [267], the International Dysphagia Diet Standardisation Initiative (IDDSI) [268], and the Japanese Dysphagia Diet 2013 (JDD2013) [269] all using different and arbitrary viscosity levels and terminology. The ESSD proposes to go further than just the use of a descriptive classification of viscosity levels and therefore recommends that manufacturers of thickening agents also include the viscosity values in Système Internationale d’Unités (SI) units (mPa·s) into the labels of thickening agents [100, 270, 271](Fig. 2). The assessment of ‘optimal’ volume and viscosity level can be done under direct view using VFSS or FEES (see "Videofluoroscopic swallow study " and "Fiberoptic endoscopic evaluation of swallowing" sections).

#### Texture modified foods (solids)

The level of scientific evidence of the therapeutic effect of texture modified foods in HNC patients is still low [272]. Several food textural and rheological properties, such as ‘elasticity’, ‘hardness’, ‘gumminess’, ‘springiness’, ‘creaminess’, ‘crispness’, ‘brittleness’, ‘chewiness’, ‘adhesiveness’, and ‘cohesiveness’, are used to describe solid food boluses. A texture modified diet should include both a nutritional adaptation (caloric and protein content) and an organoleptic adaptation (smell, taste, appearance, etc.) [261]. It is argued that the clinical strategy called ‘The Minimal-Massive Intervention’ (MMI) provided strong clinical benefits in elderly patients and could also be applied to HNC patients [273]. Before promoting the MMI in oncological populations, additional research is needed. The Test of Masticating and Swallowing Solids (TOMASS) was developed as a quantitative assessment of solid bolus ingestion [274]. It measures the number of masticatory cycles and swallows per bite, and time per bite, masticatory cycle and swallow [274]. The TOMASS was not validated to prescribe the optimal solid texture level but it can certainly contribute to making a patient-tailored clinical OD management plan for solid bolus ingestion.

#### Hidden risks of bolus consistency modification

The adherence to thickeners in general and XGB thickeners in particular is very low due to unpalatability leading to an increased risk of dehydration [262, 275, 276]. Furthermore, the exclusion of some solid foods due to the difficulty to adapt them to an optimal texture might decrease the nutritional content of meals [277]. Furthermore, patients treated with (C)RT may have difficulties with texture modified foods, as the taste and texture of modified foods can be less well tolerated by HNC patients with nausea, taste disturbances, mucositis, oral candida, and xerostomia [278]. Hydration and nutrition monitoring as well as clinical supervision of the potential respiratory tract complications of OD are essential for patients requiring bolus consistency modification (see  "Complications due to oropharyngeal dysphagia in head and neck cancer", "Cancer cachexia in head and neck cancer", and "Treatment of cancer cachexia" sections).

### Head and neck cancer specific exercise treatment for oropharyngeal dysphagia

The goal of swallowing exercises is to change swallow physiology through targeting strength and/or range of movement of muscles and to improve sensory feedback responsible for motor programming and execution to improve swallowing safety and efficiency. Several different exercises are used in clinical practice targeting the oral tongue, lips, base of tongue, jaw, soft palate, larynx, and pharynx. Swallowing exercises are differentiated from compensatory strategies that alter swallowing and facilitate improvement whilst an individual is eating or drinking. The use of compensatory strategies such as a chin tuck, head turn, or supraglottic swallow may immediately facilitate safer swallowing during a given moment, but are unlikely to create lasting changes to the physiology of the swallow mechanism [279]. Swallowing exercises were traditionally provided as part of rehabilitation after completion of all HNC treatment, although recent literature has questioned the benefit of these exercises provided months after cancer treatment [280]. Over the last decade, support from clinical researchers for the use of *prophylactic* swallowing exercises has grown, centered on the premise that strength-based and/or range of movement exercises targeted at the swallowing muscles may prevent muscle atrophy and reduce or delay the impact of RT-induced fibrosis [69, 281–283]. Despite good biological plausibility, the most recent Cochrane systematic review found insufficient high-quality empirical evidence to support the routine use of prophylactic swallowing exercises [284]. A separate review [57] using a health behavior change model to analyze a similar body of evidence to the Cochrane review questioned whether other factors such as patient adherence to exercises may be contributing to poor results in efficacy trials of prophylactic swallowing exercises. The findings from both these reviews call for larger, methodologically robust trials that address previous shortcomings such as poor patient adherence to exercises and the type and timing of outcomes. Thus based on the currently existing evidence, it remains uncertain whether patients who undertake prophylactic swallowing exercises experience better swallowing outcomes after HNC treatment compared with those who do not.

A meta-analysis review exploring OD treatment across the pathway of care for people with HNC found evidence for some improvements to selected aspects of swallowing function and jaw opening for exercises before and after oncology treatment, but no evidence for improved QOL [285]. These authors likewise advised on the need for further well-designed trials. Since these reviews, a few randomized clinical trials of swallowing exercise interventions have been published and/or are currently underway each with differing foci. These include a theory-based behavior change approach to swallowing exercise interventions [286], prophylactic exercises delivered via different methods including home-based, home-based with counseling via an App, face-to-face therapy [287], and swallowing exercises with respiratory muscle training randomized to two groups of patients; before CRT or immediately after treatment completion [288].

It is clear from the above, that there are a number of rehabilitation/prehabilitation programs available [285] and several novel developments on the horizon for which a useful overview is provided by Cuicci and colleagues [289]. Two of the more commonly used programs include Pharyngocize [283], a standardized high-intensity swallow exercise program including exercise and diet modification administered daily by the therapist throughout the duration of (C)RT; and the McNeil Dysphagia Program [290] which incorporates principles of progressive resistance and load. A bolus driven approach is used to systematically increase oral intake across a variety of food textures whilst simultaneously training swallowing. The use of adjunctive device-driven exercise protocols that include biofeedback, such as the Iowa Oral Performance Instrument (IOPI) [291] or expiratory muscle strength training [292] may also offer some benefit. At present, we have limited high-quality evidence from empirical studies to inform practice around the best timing and the optimal dose for swallowing exercises, but research in this field is at an exciting phase and continues to incrementally improve our understanding and knowledge of the benefit of swallowing exercises in people with HNC. Furthermore, we are entering a new era of technology and practice advancement with many research innovations emerging [289]. The choice of potential exercise interventions for clinicians working with this population of patients is therefore quite varied. Given the rapidly changing landscape within this field, it is important that decisions are made on the basis of best available evidence together with what is most feasible and acceptable for individual patients within the context of their care.

### Oral health in head and neck cancer

Prevention and treatment of dental and jaw-related problems deserve special attention as they affect the health of HNC patients in multiple dimensions including the swallowing function. Osteoradionecrosis (ORN) is one such problem which causes significant morbidity, even though a decreased incidence is reported since the introduction of IMRT [293]. ORN usually presents as exposed necrotic bone, ulceration, suppuration, pain, lymphadenopathy and/or paresthesia [294]. The mandible is most commonly affected due to the dense bone and its vulnerable blood supply [295]. HNC patients who develop ORN have usually received a RT dose of 60 Gy + [296]. A systematic review found that while the incidence of ORN after post-irradiation tooth extractions is low, the extraction of mandibular teeth within the radiation field in patients who received a radiation dose higher than 60 Gy represents the highest risk of developing ORN [296]. In the majority of cases, ORN develops within the first 3 years (74%) after RT but it can also occur long-term post-RT. Tooth extraction causes approximately 50% of the ORN cases and 30% occur spontaneously following HNC treatment [296, 297]. Risk factors for ORN include trauma from dental extractions, ill-fitting dentures, and periodontal infection affecting the tooth furcation and alveolar bone [298]. The furcation is situated between the roots of posterior teeth, normally covered in bone. Furcation exposure due to periodontitis may result in infection in this difficult to access area. Monitoring and early intervention to maintain oral health is advised with a careful periodontal or radiographic examination. Treatment modalities for ORN range from conservative (oral rinses, irrigation, and antibiotics) to surgical resection of the jaw depending on the extent and severity of the problem [294, 299]. The use of hyperbaric oxygen may be considered, but supporting evidence is weak [300, 301]. Following RT, dental extractions from the bone which has received high dose radiation should only be undertaken if unavoidable—endodontic treatment and decoronation should be considered first [302]. In the context of oral health and dental rehabilitation, treating periodontal disease and restoring occlusal function are important for mastication of food and bolus preparation, facilitating nutritional intake, and reducing the risk of aspiration of food and/or of oral micro-organisms into the respiratory tract [303–306].

Dry-mouth may compromise oral health in this population. It affects up to 85% of HNC survivors following RT with the salivary glands in the field of radiation [307, 308]. Dry-mouth and poor compliance with regular dental attendance, OH, diet, and daily fluoride use are significantly associated with the development of dental decay requiring tooth extraction following HNC treatment [309]. ORN prevention is therefore an important point of attention and is preferably initiated with oral care pre-(C)RT [310, 311]. An interesting but not unexpected development is that the reported incidence of ORN has decreased from 20–30 to 4–8% in recent studies using IMRT [312]. In addition to IMRT positioning or shielding stents can also protect healthy tissues, including salivary glands, alveolar bone, and masticatory muscles [302]. Pre-RT extraction of teeth of questionable prognosis in the high-dose RT field is recommended [313, 314]. Healthy and unerupted teeth covered by bone are usually retained [313]. Decisions regarding extractions also require an assessment of the likely future compliance of the HNC patient with oral care [299, 309, 315]. If there is no residual salivary gland function, salivary substitutes can be explored for oral-lubrication to reduce oropharyngeal dryness and improve HRQOL [316]. If there is some residual function, salivary gland stimulants (e.g. sugar-free chewing-gum, pilocarpine/cevimeline) may give some relief [317, 318]. It is recommended that dentate patients with HNC avoid saliva-stimulating products containing citric acid because it can support dental erosion. A number of HNC patients also report long-term preference for plain tap water as saliva substitutes provide only short-term relief [315]. A high energy, carbohydrate diet may cause rampant dental decay due to the dry-mouth related shift to cariogenic oral microorganisms. It is strongly recommended to avoid sugar in the diet, use toothpaste that has a higher fluoride concentration (4%) and to carry out meticulous plaque removal long-term post-RT in HNC patients to prevent further dental decay [319, 320]. Attention should also be paid to special situations such as limited access to posterior teeth due to trismus arising from reduced flexibility or fibrosis of masticatory muscles and buccal mucosa following (C)RT.

Excellent OH is recommend using a small-headed pediatric, end-tufted or interdental toothbrush, floss/airflosser, and Waterpik® as interdental food stagnation is common with dry-mouth, increasing the risk of dental decay and food/bacteria aspiration [304, 305, 319, 321]. Products like chlorhexidine liquid (0.2% or 0.1%) may be used as a brush-on to reduce staphylococcus mutants levels and control gingival inflammation [322, 323]. Careful subgingival scaling and furcation monitoring during oncological follow-up are necessary to lower the risk of ORN [316].

Oral function of the HNC survivor should be carefully assessed post-surgery/RT to determine the need for dental rehabilitation. A shortened dental arch (SDA) with 20 functioning units seems to maintain masticatory function, aesthetics, satisfaction, psychosocial ability, occlusal support, jaw stability, tactile perception, speech-articulation, and taste [324]. It is suggested that patients with a SDA have an acceptable level of OH-related QOL [325]. Dentures should be made carefully with stability, retention, and load distribution across the tissues as surgical defects and RT-induced fibrosis increase the complexity of denture design [326, 327]. Osseointegrated dental implants can be life-transforming for HNC survivors suffering from dry-mouth or following major surgical resection, but studies suggest that implant survival is strongly influenced by RT. Successful implant placement into irradiated bone was reported by Pompa and colleagues [328]. However, 76% of HNC irradiated patients in this study received 36 Gy with only 24% receiving 60 Gy. Significantly better implant survival in non-irradiated bone has been reported [329]. Optimal timing of implant placement in HNC patients planned for (C)RT is controversial. Implants may be placed pre-RT at the time of cancer surgery or post-RT [330]. Implant placement at the time of surgery facilitates osseointegration pre-RT reducing the risk of late complications (e.g. ORN) [303]. However, the risk of inappropriate implant positioning during oncological surgery has been described making subsequent prosthodontic treatment more complex [331]. A review comprising 416 patients who had a fibula-free-flap reconstruction following segmental mandibulectomy reported that postoperative RT was a contra-indication for dental implants due to the high risk of ORN [299]. Furthermore, the authors concluded, “*secondary dental implant placement involves multiple surgeries, hospitalizations, and financial burden. Primary implant placement during the time of tumor resection, either in native or reconstructed non-irradiated bone, offers an opportunity for implant supported restorations to the oral cancer patient*” p.1734 [299]. Finally, it is recommended to discuss dental rehabilitation and long-term OH early during MDT meetings and subsequent treatment decisions should be made on a case-by-case basis [315].

### Treatment of trismus following (chemo)radiotherapy

The normal range of Inter-incisal Mouth-Opening (IMO) is 40–60 mm. Mouth opening restricted to 35 mm or less is considered to be trismus [332, 333]. Pre-RT IMO is a significant indicator for the development of trismus post-RT [334]. Trismus is related to masticatory muscle damage, temporomandibular articulation (TMA) damage, fibrous ankylosis and/or rapid growth of scar (connective) tissue. This condition can be very painful, resulting in a vicious-cycle where more rapid and forceful attempts at mouth opening can generate more reflex contraction thus generating increased pain [335]. However, also without any movement, a degenerative TMA may occur with muscle atrophy and shortening of masticatory muscle fibers [336]. In assessment, reduction of mouth opening due to tumor recurrence should always be excluded first [337]. The prevalence of trismus in HNC patients is reported at 25.5% following conventional RT versus 5% after IMRT and 30.7% using (C)RT and 3D-CRT [338]. The prevalence of trismus increases with increasing doses of RT to the masticatory structures [337]. It has been concluded that novel RT modalities may decrease the prevalence and severity of trismus compared to conventional RT [334, 337]. However, despite better-focused RT dose and improved screening, progressive masticatory muscle stiffness and limitation of mouth opening remain a common complication [339]. It is suggested that limiting dose to these structures to ≤ 40 Gy for tumors not invading the masticatory muscles may improve treatment-related sequelae [340].

Limited mouth-opening directly affects many aspects of daily life such as taste, salivation, mastication, swallowing, eating, dental health, pain in head and neck, speech, voice, communication, etc. [341]. Trismus can have serious health and HRQOL implications, including those of nutritional nature due to impaired mastication if food is not properly broken down also posing an aspiration and choking risk during swallowing (see "Cancer cachexia in head and neck cancer", "Bolus consistency modification", and "Treatment of cancer cachexia" sections). Furthermore, difficulty during speaking, compromised OH and delivery of dental care are other consequences of trismus [335, 342, 343]. Mouth opening post-RT decreases on average by approximately 20% compared to mouth opening pre-RT [337]. Regular measurements of maximal mouth opening are needed to predict the development of trismus [344]. The risk of developing post-RT trismus can be related to a variety of factors including tumor location, RT dose to the primary tumor, RT dose to the muscles of mastication, age, gender, and pre-RT mouth-opening measurements [344].

Early intervention is essential to prevent long-term disability [337, 345]. The prevention of trismus, rather than its treatment, is the most important objective [337, 346, 347]. There should be regular post-RT reviews and measurement of IMO with dental or SLP professionals. Treatment of trismus can be conservative (with either medical or physical therapy) or surgical. Exercise therapy is the mainstay of treatment and should start as soon as possible after (C)RT. Prosthetic RT devices, including positioning and shielding stents, may be considered to reduce RT-induced tissue morbidity [302]. Early trismus management using exercise, physiotherapy, massage, slow motion, and wide movements avoiding pain, wood-sticks or TheraBite^®^ is recommended. The use of structured exercise with the jaw-mobilizing device TheraBite^®^ seems to be beneficial for patients with trismus independent of time since the oncological treatment [348]. Exercises should be performed intensively on a daily basis. The aim is to provide a better oral preparatory swallowing phase whilst improving speech and articulation [283, 349]. Passive motion applied several times per day is suggested as more effective than static stretching providing a significant reduction in inflammation and pain. Ongoing exercise during the HNC person’s lifetime is recommended since fibrosis will continue to progress as soon as exercises stop [283, 336, 342, 349, 350]. A systematic review of the effects of exercise therapy for trismus secondary to HNC reported considerable differences in mouth opening ranges and no evidence that any stretching technique was superior for either prevention or treatment of trismus [351]. Other interventions that decrease the severity of cancer treatment-related trismus include pentoxifylline to improve microcirculation and tissue oxygenation [352]. However, reported compliance with pentoxifylline appears to be limited by the effects of nausea [353]. Botulinum toxin injection may be used to reduce pain associated with trismus, although it does not improve jaw mobility [354]. Coronoidectomy may be used to increase the range of jaw motion and mouth opening [355]. However, controlled studies on the efficacy of these interventions are lacking in this area [338].

### Surgical treatment of oropharyngeal dysphagia in head and neck cancer

As described in the previous sections of this paper, OD in HNC patients may be the result of alterations in the upper aerodigestive tract caused by the disease and/or oncological treatment modalities. Dysfunction of the UES is often multifactorial in HNC patients and may be due to (C)RT, pharyngeal closure technique and the extent of additional pharyngeal mucosa resection in case of TLE, and postoperative complications [69, 356–358]. The incidence of cervical esophageal strictures following HNC treatment varies between 5 and 15% [359–361]. Since 1951, studies have reported several surgical and less-invasive treatments for UES dysfunction. The effectiveness of each treatment in the management of OD, due to various etiologies including HNC and its oncological treatment, is still subject to debate [362–365]. A malfunctioning UES can be diagnosed as described under previous sections of this paper (see  "Videofluoroscopic swallow study" and "Manometry"). To date, there are too few large studies describing the indications, side effects, and results of the different surgical modalities for UES dysfunction in HNC patients. The Dutch national guideline on OD was published in 2017 [365]. The guideline was developed using robust methodological techniques [366] and the studies on surgical treatment for OD were obtained using separate systematic literature searches on the following: myotomy of the UES, botulinum toxin injection in the UES, and dilatation of the UES or proximal esophagus strictures. All studies on myotomy and botulinum toxin injection in the UES were excluded based on methodological quality. For evidence on dilatation of the UES, only one article was judged to have sufficient methodological quality [367]. This study investigated non-HNC patients with OD following dilatation of the esophagus by means of a “through-the-scope balloon”. However, these results cannot simply be extrapolated to a HNC patient population and further research with HNC populations is required. Recent systematic reviews confirmed these findings of insufficient evidence to guide clinical practice for HNC patients [362–364]. Despite the uncertain evidence, myotomy of the UES, botulinum toxin injection in the UES, and dilatation of the UES or proximal esophagus strictures remain frequently applied surgical modalities in the management of OD in HNC patients following oncological treatment [363, 365].

Treatment of HNC with (C)RT can result in strictures of the cervical esophagus, often at the level of the cricopharyngeal segment [368]. UES myotomy is one of the main surgical options for the treatment of OD in HNC. It can be performed transorally or via an external transcervical approach. Transoral CO2 laser-assisted myotomy is typically preferred because it is deemed “simpler”, safer and less invasive than the external approach and is considered as effective as an external myotomy [369]. All muscle fibers are cut using the CO2 laser until the buccopharyngeal fascia is reached. Some surgeons leave the wound open [362], others apply fibrin glue [368] or suture the mucosa over the incised muscle fibers to prevent salivary leakage causing mediastinitis [370]. If endoscopic myotomy cannot be performed due to trismus or pharyngeal strictures an external myotomy can be considered. A left-sided external approach is preferred because the left recurrent laryngeal nerve is longer than the right and has a more complex route, making it more prone to injury. However, if unilateral vocal fold paralysis already exists, it is wise to select that paralytic side to avoid the paralysis of the healthy vocal fold. Despite the lack of high-quality studies, there is some evidence to justify the use of UES myotomy in HNC patients. UES myotomy significantly improved swallowing outcomes of HNC patients with OD following organ preservation therapy [362]. Research by Silver and colleagues also revealed recovery of OD in 90% of their HNC patients following CO2 laser myotomy of a post-(C)RT stenosis at the UES level [368].

Postoperative swallowing rehabilitation is recommended [364]. However, as described in previous sections of this paper, it seems less likely that OD in HNC patients is only the result of an isolated UES dysfunction. There is certainly literature available that can support the use of such surgical UES interventions in well-selected HNC patients. We, therefore, recommend that the indication for surgery should be based on a robust swallowing assessment using at least an instrumental assessment tool as described under "Videofluoroscopic swallow study ", "Fiberoptic endoscopic evaluation of swallowing", and Manometry" sections, a patient self-report tool (see " Screening and clinical assessment of oropharyngeal dysphagia in head and neck cancer" ), and a nutritional assessment (see  "Cancer cachexia in head and neck cancer" and "Treatment of cancer cachexia" sections). Such a multi-dimensional swallowing assessment can help in predicting the chance of success of a particular surgical modality for a patient and is also essential in the risk–benefit estimation of such interventions. Adverse events following UES surgical interventions, such as severe recurrent aspiration pneumonia due to severe gastroesophageal reflux following UES myotomy in the presence of a dysfunctional lower esophageal sphincter have been reported [371, 372]. The most important adverse event of such UES interventions is an esophageal perforation accompanied by mediastinitis, sepsis, and a high risk of death [363]. Fortunately, the chance of such an adverse event is low in the hands of experienced surgeons and carefully selected patients. Surgical interventions have also been described for HNC patients with very severe and difficult-to-treat aspiration. In particular, patients with severe OD-related respiratory tract complications may benefit from surgical interventions such as vocal fold medialization techniques in case of vocal fold paralysis [373] (see "Management of oropharyngeal dysphagia as an adverse event and in case of palliation" section), a tracheotomy using cuffed tracheostomy tubes (see "Tracheostomy and oropharyngeal dysphagia in head and neck cancer" and "Complications due to oropharyngeal dysphagia in head and neck cancer" sections), laryngeal suspension surgery [370, 375, 374], and in selected cases laryngotracheal separation or a TLE [376–379]. These are, of course, interventions that are considered 'last resort surgical interventions' for the treatment of severe life-threatening OD. The number of studies on laryngeal suspension surgery and laryngotracheal separation in HNC patients is very limited and it mainly concerns smaller case series with a low level of evidence [370, 374–376]. The body of literature on functional laryngectomy for end-stage OD in HNC patients is slightly more extensive [377–379]. These surgical interventions do not guarantee an overall improvement of the swallowing function nor a normalization of oral intake without feeding tube dependency. Again, it is essential to make a multidisciplinary risk–benefit estimation of such interventions and to discuss the adverse events with the patient during informed consent.

### Treatment of cancer cachexia

The multifactorial underlying mechanism of cancer cachexia requires multimodal treatment strategies. The main challenge for HNC patients is meeting sufficient oral intake requirement levels. Different HNC treatment modalities require different nutritional approaches [380, 381]. Postoperatively a nasogastric feeding tube (NGT) is often inserted for a short period of time (10 days) to optimize wound healing and to bridge a period of OD and/or odynophagia. Nutritional guidance during (C)RT of locally advanced head and neck tumors is more complex since maintaining sufficient oral intake is challenging due to high toxicity rates. Side effects, including, amongst others, mucositis [382], xerostomia, sensory changes, pain, OD, nausea, and vomiting [383, 384] lead to temporary tube feeding (TF) dependency in 37–74% of the patients undergoing CRT [385–387]. Maintaining good nutritional status and preventing weight loss have been associated with improved treatment tolerance, reduced risk of complications and associated therapy delay, increased response rate to (C)RT and higher survival rates [388–390]. Additionally, taking into account the phenomenon of sarcopenic OD, accurate nutritional support is of utmost importance. The additional value of dietary counseling and nutritional support in HNC is clear [391]. However, the optimal type of tube (NGT or a gastrostomy) and timing of insertion (prophylactically or reactively) in HNC patients undergoing (C)RT remains controversial since high-quality randomized trials are lacking [392]. NGTs are often considered to be uncomfortable, impeding oral intake due to pharyngeal irritation, and can be associated with reduced HRQOL compared to gastrostomy [393, 394]. Therefore, a gastrostomy is considered superior to a NGT when TF is required for a longer period of time. Gastrostomy tube insertion is recommended when TF extends beyond the expected use for 4–6 weeks [381, 395, 396]. Gastrostomy tubes may be inserted before the start of concurrent (C)RT (i.e. prophylactically). Having a gastrostomy in situ can facilitate immediate initiation of TF when necessary and enables direct administration of fluids in case of nephrotoxicity. Additionally, this timing prevents the risk of treatment-induced neutropenia or mucositis complicating tube insertion. This immediate action could prevent further weight loss and organ damage. In addition, a better patient adherence to prophylactic TF might be expected compared to reactive feeding tube placement [397]. However, a gastrostomy is accompanied with complication rates of about 3.3–19% [398, 399] and between 9 and 47% of the prophylactic gastrostomies are never used [387, 400]. Furthermore, prophylactic gastrostomy insertion has been argued because it might lead to long-term OD, considering the “use it or lose it” principle regarding the swallowing mechanism [336, 401] but high-quality randomized trials to prove this theory are not available and other studies have shown no relationship between prophylactic gastrostomy and long-term swallowing function [402, 403]. Right now, high-quality evidence on the advantage of prophylactic gastrostomy placement in HNC patients undergoing (C)RT is lacking and routine prophylactic gastrostomy in all these patients is certainly not advised. However, pre-(C)RT prophylactic gastrostomy placement in patients with a high risk of or pre-existing malnutrition and OD seems to be of added value, so that their survival will not be compromised by interrupting the (C)RT for reactive gastrostomy placement. Prediction models to identify patients requiring prophylactic gastrostomy insertion are currently under development [387, 404].

Catabolic effects on the skeletal muscles and potentially also on swallowing muscles in cancer cachexia, are not entirely reversible using conventional nutritional support. Besides, providing sufficient calories and high-quality proteins, specific nutrients including eicosapentaenoic acid (EPA) and vitamin D have been investigated for their added value in cancer cachexia and previous pilot studies warrant further investigation in appropriately powered randomized clinical trials [405–407]. Timing of targeted nutritional supplementation is also important to consider to stimulate synergistic nutritional effects but to avoid adverse events from (C)RT [408]. More attention should be paid to optimize the nutritional status during the pretreatment as well as the recovery phase.

Next to nutritional support, skeletal muscle stimulation is important to combat systemic muscle catabolism. In HNC patients, resistance training of several skeletal muscles appeared feasible during (C)RT [409, 410]. Due to small study populations, the evidence is not clear on whether this resistance training actually leads to improved survival rates, but there seems to be a positive effect on HRQOL. Ideally, the physical exercise program is integrated into standard care to increase patient adherence to exercise. In case of functional deterioration during (C)RT, which complicates physical capacity, neuromuscular electrical stimulation might be considered as an alternative intervention for resistance exercises [411] but requires further investigation and specification of skeletal muscle groups.

### Management of oropharyngeal dysphagia as an adverse event and in case of palliation

The tumor itself may cause impaired swallowing function or impaired bolus passage, but swallowing can also be affected by adverse effects of the HNC treatment (surgery, RT, CRT, or combinations thereof—multimodality treatment) (see Pathophysiology and mechanisms of oropharyngeal dysphagia). OD with and without aspiration may occur due to changes in the anatomy, tissue properties, and sensory input of the oropharyngolaryngeal tract (cranial and cervical nerves VII, IX-XII, V2, V3, C1 C2) [69, 412, 413]. The extent of the malfunction depends on the size and location of the tumor as well as on the resected area, the reconstruction method, the extent of sensory reinnervation, and the application of postoperative (C)RT [414, 415]. For example, maxillary sinus tumor excisions and velar surgery may leave oronasal or oromaxillary fistulae affecting oral bolus containment, velopharyngeal junction closure, pharyngeal pressure generation, and speech production. Intraoral prosthetic rehabilitation is often beneficial in this context and may improve the impaired oral bolus containment, velopharyngeal junction closure, pharyngeal pressure generation, and speech production [292, 417, 416]. Also therapy-induced facial nerve paresis, for example as an adverse event following an extended parotidectomy or excision of a skull-base or temporal bone tumor, may result in impaired stomatognathic functions, such as mastication, swallowing, and suction. Neuromuscular re-education exercises, surface electromyographic biofeedback (sEMG), chemodenervation or dynamic surgical facial reanimation in case of a sacrificed facial nerve may be beneficial [418]. Neck dissections sacrificing the hypoglossal nerve, mandibular branch of the facial nerve, mylohyoid branches of the trigeminal nerve or the auricular branch of the facial nerve may also result in OD often with aspiration. Supraglottic laryngectomy extending to tongue base or arytenoid level is also accompanied by an increased risk of aspiration [419]. Standard pre- and post-oncological treatment assessment of swallowing to identify the nature and severity of OD as described in "Videofluoroscopic swallow study ", "Fiberoptic endoscopic evaluation of swallowing", and Manometry" sections are recommended in the context of best practice [88] and OD interventions are often indicated (see "Bolus consistency modification" and "Head and neck cancer specific exercise treatment for oropharyngeal dysphagia"). Nowadays, in the case of (C)RT for advanced HNC, IMRT appears to be significantly beneficial in sparing swallowing function without compromising survival due to dose reduction to swallowing structures, as described in "Pathophysiology and mechanisms of oropharyngeal dysphagia", Oral health in head and neck cancer" and " Treatment of trismus following (chemo)radiotherapy", [420]. In addition, acute radiation-associated dysphagia (ARAD) also exists and is well documented in the literature [357]. Persistent or late OD-inducing conditions comprise ORN, dental decay, trismus, xerostomia, stenosis, fibrosis, etc. The characteristics of ARAD may include silent aspiration and pharyngeal residue [69]. An intensive ‘boot camp’ type approach to treatment using sEMG biofeedback and bolus-driven therapy such as the McNeil Dysphagia Program has been described in Head and neck cancer specific exercise treatment for oropharyngeal dysphagia" section. In case of severe life-threatening posttreatment intractable aspiration, surgical options to separate the alimentary and respiratory tracts may be considered [421] (see "Surgical treatment of oropharyngeal dysphagia in head and neck cancer section). Furthermore, as vocal fold paralysis from vagal nerve damage may contribute to aspiration risk as well [422], posttreatment vocal fold medialization techniques to compensate the vocal fold closure gap may reduce aspiration [423] (see "Surgical treatment of oropharyngeal dysphagia in head and neck cancer" section). Vocal fold augmentation using hyaluronic acid under topical anesthesia to facilitate laryngeal closure can also be considered for the palliative patient population, as this may improve respiratory tract clearance, reduce aspiration risk, improve voice function, and HRQOL [424]. In addition, more permanent solutions such as laryngeal framework surgery or permanent injection augmentation with material such as polydimethylsiloxan may be considered in the context of palliative treatment too. Furthermore, trismus is a common adverse event following oncological treatment of the area of the masticatory structures and rehabilitation is very relevant in the HNC population and described in " Treatment of trismus following (chemo)radiotherapy" section. In general, palliative OD rehabilitation focuses on the recovery of dignity, facilitating the person to have control within their physical limitations [425]. Several interventions described above can also be of added value for HNC patients in a palliative care setting. Instrumental swallowing assessment remains of great importance in the palliative HNC patient population as well to guide decision-making for HRQOL and to support patient autonomy (see "Videofluoroscopic swallow study ", "Fiberoptic endoscopic evaluation of swallowing", and Manometry" sections). Following informed consent where patients acknowledge risks associated with oral intake, the palliative or non-palliative HNC patient may decide to continue to eat and drink without any restrictions as described in "Ethical issues, informed consent, and the right to refuse oropharyngeal dysphagia treatment" section. Along with standard interventions, fastidious OH may reduce aspiration pneumonia and enhance HRQOL (see "Complications due to oropharyngeal dysphagia in head and neck cancer" and "Oral health in head and neck cancer" sections) [426]. Finally, palliative TF or enteral feeding and a palliative tracheostomy clearly have a place in the care offered for these patients and are not necessarily contraindicated (see "Ethical issues, informed consent, and the right to refuse oropharyngeal dysphagia treatment" section).

### Oropharyngeal dysphagia following total laryngectomy

TLE is a surgical procedure that consists of removing the larynx and separating the airway and digestive tract. It is used as a treatment for advanced-stage laryngeal and hypopharyngeal cancer. If necessary, a TLE can be carried out in combination with a partial or total pharyngectomy or even with an esophagectomy. In the case of the latter two, a neopharynx is created by closing the pharyngeal defect with different types of pedicled or free flaps or with a gastric pull up. Preservation of swallowing and restoration of speech are important additional goals. OD is a common symptom following TLE and its frequency ranges from 10 to 72% [427–429]. The main complaints reported by TLE patients are regurgitation even into the nasal cavity, food sticking in the throat, pharyngeal globus sensation, or a prolonged mealtime [357, 430, 429]. The development of OD following TLE may be caused by multiple factors such as RT (primary (C)RT followed by salvage TLE or TLE with postoperative (C)RT), the closure technique to create a neopharynx, the extent of additional pharyngeal mucosa resection, and postoperative complications [357, 430–432]. Swallowing assessment in TLE patients can be carried out using instrumental tools as described in "Videofluoroscopic swallow study ", "Fiberoptic endoscopic evaluation of swallowing", and Manometry" sections. Swallowing impairment should also be evaluated from the patients’ perspective using self-report OD questionnaires. The Swallowing Outcome After Laryngectomy questionnaire (SOAL) has been validated specifically for patients without a larynx [432, 433]. Such questionnaires may be a useful way of monitoring swallowing and signposting the clinician toward the need for further diagnostic swallowing assessments. A recently published systematic review suggests that the most frequently identified functional and/or structural alterations causing OD are weakness of the pharyngeal constrictor muscles, increased resistance to the passage of a bolus, pharyngoesophageal strictures, and pseudodiverticulum formation [434]. It remains unclear which pharyngeal closure technique is the best for swallowing [434]. In this systematic review, it was not possible to stratify swallowing outcomes per surgical technique due to the great variation in the techniques used, for example, TLE with primary closure or TLE with partial pharyngectomy and flap reconstruction, total pharyngolaryngectomy with different flap reconstructions, gastric pull-up, TLE with or without myotomy, etc. (Neo)adjuvant (C)RT had a negative impact on swallowing although a detailed description of these non-surgical techniques was missing in the majority of the studies [435–440]. The absence of guidelines for swallowing assessment in TLE patients and the lack of validated measurements for this population might explain the great diversity of assessment tools that were applied. The body of literature on treatment effects of OD after TLE remains poor and there is no consensus on the diagnosis and treatment of OD for this population [434]. Some studies showed preliminary promising results of botulinum toxin injections, endoscopic dilatations, and TOLS [441–448]. However, significant treatment effects or trends of these techniques were not found. There is a need for well-designed trials using validated multidimensional swallowing assessment protocols to evaluate OD following TLE and to investigate the clinical applicability and effects of treatment techniques.

## Ethical issues, informed consent, and the right to refuse oropharyngeal dysphagia treatment

Beneficence, non-maleficence, justice, and autonomy are interdependent principles that govern present day clinical ethics in most countries [449]. Health professionals apply these basic principles in daily care when confronted with conflicts of values. The principles by themselves are insufficient but constitute a framework that helps us to use a rational method in reaching decisions. More often than not, resolution of ethical conflicts will occur after an interprofessional discussion involving the patient, next of kin, and legal representatives or designated persons with power of attorney. In the context of OD treatment, health professionals will face two types of situations, each with its potential ethical conflicts. In the acute situation, the clinical problem, its complexity and prognosis will be the primary drivers when considering an intervention such as artificial nutrition and hydration (ANH) typically of short duration [450]. In the context of advanced or terminal illness, patient comfort, palliation, and non-maleficence will more strongly influence clinical decision-making. Proportionality of the intervention, inconvenience and discomfort for the patient, patient preference, and the existence of advanced directives will influence the outcome. In this situation, ethical conflicts may occur and an extensive body of literature on this topic already exists as indicated by Druml and colleagues [451]. Given nutrition’s vital nature, even proposals for simple interventions to treat OD might be met with moral objections from a patient, influenced by cultural or religious factors. The abstention of ANH can elicit intense discussions between patients, but even more so between families and medical teams. Starting abstention and stopping ANH are decisions with an important ethical dimension. The Catholic Church has agreed that the abstention of ANH is not defined as euthanasia, and has recognized that in some circumstances, its medical futility can be taken into account [452]. Within the Protestant Church many divergent opinions on the subject exist. The Jewish religion distinguishes between active and passive proceedings and might permit the withholding of ANH but forbids its withdrawing. For conservative and orthodox Jews nutrition is a basic need and not a treatment. The Islam religion considers food and hydration as basic needs too and not as medical treatment but with patient or proxy consent, it can be withdrawn or withheld [451]. It is now commonly accepted that ANH is a medical intervention, with all consequences involved and therefore also needs informed consent. In the past, health professionals would intervene with whatever treatment they considered best for the patient. Fortunately, present daycare takes into perspective the personal preferences of the patient. Therefore, health professionals must obtain the patient’s (or the patient’s legal representative’s) free and informed consent on OD treatment before it commences. Only in emergencies, in non-communicative patients, and in the absence of representation by next of kin should the medical team make decisions in the best interests of the patient. Admittedly, physicians sometimes face conflicts of interest when a patient’s or family’s decision does not seem to align with the current guidelines or evidence-based medicine. This situation can occur when cultural, religious or even superstition influences the decision. The rising problem is that of language barriers and hospitals should be prepared to have interpreters on hand to help physicians and MDT members communicate with patients and relatives in many different languages. Pictorial aids could facilitate obtaining consent in children or illiterate patients. Experienced SLPs play a role in facilitating communication and should be involved at this point. Obtaining informed consent means that the health professionals must have clearly informed the patient about the characteristics of the intended intervention. Not only how information is presented is important in influencing decision-making, but also the time needed by the patient to reflect on choices. When medical conditions permit, such as in elective surgery, the information should be presented as early as possible. The future need for TF depends on the extent of the disease, type of HNC treatment, and wishes of the individual patient as described in "Treatment of cancer cachexia" section. The information that should be offered relates to the following aspects of the intervention: its purpose, nature, degree of urgency, duration, frequency, contra-indications, side effects, relevant risks, necessary aftercare, financial consequences, possible consequences of a refusal to consent, and the possible alternatives. In the majority of the European countries a legal cascade of representatives, usually a spouse or next of kin, are able to make the final decision in the absence of advance directives. More and more people now have written “advance directives”, in which they specify the type of medical care they would want to receive in a particular condition and certainly when he or she would no longer be able, due to incapacity, to make such decisions, and stipulate who should make those decisions for them. In this “living will” people can be granted a durable power of attorney. Health professionals should realize that OD is not merely the impossibility to ingest food through the mouth to the stomach. Eating is not merely the oral ingestion of food, followed by several physiological processes that result in the production of vital energy. Due to its existential nature, being unable to eat or drink has a profound impact on the patient (see "Psychosocial impact" section). Therefore, OD is accompanied by a significant loss of HRQOL. Caregivers often feel morally obliged to start TF, because failing to do so would be ‘starving’ the patient. This train of thought is understandable, however, sometimes the patient benefit of TF is not always clear and the health professional should review the benefit in the short, middle, and long term, as well as possible complications, the current HRQOL, and how it can be improved by TF. A myth that should be dispelled is that the patient who does not want TF is not a candidate for swallowing intervention. Even in case of refusal of TF, swallowing evaluation and intervention should identify strategies to promote the safest means of oral intake.

## Head and neck cancer patient perspective

People treated for HNC are usually given information about how eating and drinking may be affected, but it is difficult for health professionals to adequately convey the reality of what it feels like to be a “patient with OD”. Recently, Checklin and colleagues published a study on HNC patients’ perspectives on OD care experience [453]. They emphasize that patients’ perspectives are valuable to increase insight into how patients experience their ‘rehabilitative journey’. Swallowing is something that we take for granted as human beings until it is impaired through illness. The subsequent impact is not only on our ability to obtain nutrition, but also on socializing, relationships, confidence, and attitude to “beat cancer”. Importantly, “survivorship is not survivorship without QOL”. Patient support groups have been defined in the literature as “a group of people with common experiences and concerns who provide emotional and moral support for one another,” and may also have a role in patient education, improving public awareness, and fundraising [454]. As a primary function, a support group is a forum where newly diagnosed patients, or indeed patients at any stage along their journey may seek the help and support of someone with the shared experience. This extends to families and caregivers who should also form part of this support network. It is important to recognize that support is not unidirectional, and that “veteran” members of a support group also benefit from the process. Support groups may meet face-to-face, via telephone support lines or have online forums, all options showing a positive impact on QOL for patients who are involved and engaged in giving and receiving support [455, 456]. Health professionals should be familiar with the support groups within their local geographic area as well as the most suitable online forums. All patients with HNC should be offered the opportunity and provided with details to contact a support group at the time of diagnosis. Depending on individual hospital practice, many support groups offer for one of their members to meet with patients prior to treatment, a model often used prior to TLE but potentially useful for other types of surgery too. The Swallows Head & Neck Cancer Charity (https://www.theswallows.org.uk) is one example that offers support and information to both patients and caregivers in many forms; including a 24/7 telephone support line, regular face-to-face monthly meeting groups, and an online social media presence providing topical information relevant to HNC patients. The Swallows Charity aims to function as an “umbrella” organization helping to set up local groups throughout the UK and Europe. Thus far, the Charity has eight support groups based around England and Ireland. The Charity is looking to expand its groups in Spain, Italy, and across Europe during 2020. The Charity Patient/Carers Book is in its 2nd edition and distributed in the UK [457] and Australia [458] and its 1st edition was launched in Spain [459]. What is important is that health professionals are able to signpost patients to these options. Collaboration between health professionals and patient support organizations facilitates the best care and optimal QOL for patients.

## Conclusion and topics for future research on oropharyngeal dysphagia in head and neck cancer

This European White Paper summarizes current evidence-based literature on OD in HNC and provides recommendations to support patients and health professionals. Experts from the ESSD, the CEORL-HNS, the EHNS, the UEP, and the ELS described the state of the art of various diagnostic examinations and treatments for OD or OD-related issues in HNC. The body of literature and its level of evidence on diagnostics and treatment remains poor. For some sections of this paper, it was very difficult to come up with a recommendation based on scientific evidence. Therefore, in these sections we provide expert opinion to help health professionals, caregivers, and patients with multiple challenges due to OD in HNC. Despite the fact that the scientific evidence is very limited, it remains challenging to obtain research funding in the European Union for studies on OD in HNC. Possible reasons are that there is increased competition from subjects or conditions with a much higher prevalence than HNC, for example, stroke. Research grants also tend to focus more on the treatment of cancer itself than on the functional impairments or consequences of HNC or its treatment. Yet, it is important to note that survival for this population has not increased spectacularly in recent decades and approximately 50% of the HNC patients in the long term die as a result of the disease. The question, therefore, arises as to whether grant funding agencies should pay more attention to HRQOL in the context of OD in HNC.

We anticipate an increase in the prevalence of OD due to HNC in the near future. Amongst other factors, further aging of our European population (including HNC patients) and an increase in the prevalence of HPV-related cancer, although the HPV vaccination was introduced in various countries, will contribute to this increase in HNC-related OD prevalence. We conclude with a call for further implementation of OD screening in HNC patients and emphasize that the need for scientific research on OD treatment remains a key challenge for European professional associations and policy makers.

## References

[1] Pezzuto F, Buonaguro L, Caponigro F (2015). Update on head and neck cancer: current knowledge on epidemiology, risk factors, molecular features and novel therapies. Oncol.

[2] Ferlay J, Steliarova-Foucher E, Lortet-Tieulent J (2013). Cancer incidence and mortality patterns in Europe: estimates for 40 countries in 2012. Eur J Cancer.

[3] Hashibe M, Brennan P, Chuang SC (2009). Interaction between tobacco and alcohol use and the risk of head and neck cancer: pooled analysis in the international head and neck cancer Epidemiology consortium. Cancer Epidemiol Biomarkers Prev.

[4] Orlandi E, Alfieri S, Simon C (2019). Treatment challenges in and outside a network setting: head and neck cancers. Eur J Surg Oncol.

[5] Boscolo-Rizzo P, Zorzi M, Del MA (2018). The evolution of the epidemiological landscape of head and neck cancer in Italy: Is there evidence for an increase in the incidence of potentially HPV-related carcinomas?. PLoS ONE.

[6] Mehanna H, Paleri V, West CML, Nutting C (2010). Head and neck cancer - Part 1: Epidemiology, presentation, and prevention. BMJ.

[7] Wagner S, Wittekindt C, Sharma SJ (2017). Human papillomavirus association is the most important predictor for surgically treated patients with oropharyngeal cancer. Br J Cancer.

[8] Trizna Z, Schantz SP (1992). Hereditary and environmental factors associated with risk and progression of head and neck cancer. Otolaryngol Clin North Am.

[9] Yarbrough WG, Aprelikova O, Pei H (1996). Familial tumor syndrome associated with a germline nonfunctional p16(INK4a) allele. J Natl Cancer Inst.

[10] Jefferies S, Eeles R, Goldgar D (1999). The role of genetic factors in predisposition to squamous cell cancer of the head and neck. Br J Cancer.

[11] Sun S, Pollock PM, Liu L (1997). CDKN2A mutation in a non-FAMMM kindred with cancers at multiple sites results in a functionally abnormal protein. Int J Cancer.

[12] Giraldi L, Leoncini E, Pastorino R (2017). Alcohol and cigarette consumption predict mortality in patients with head and neck cancer: a pooled analysis within the International Head and Neck Cancer Epidemiology (INHANCE) Consortium. Ann Oncol.

[13] Deleyiannis FWB, Thomas DB, Vaughan TL, Davis S (1996). Alcoholism: independent predictor of survival in patients with head and neck cancer. J Natl Cancer Inst.

[14] Smith WH, Luskin I, Resende Salgado L (2019). Risk of prolonged opioid use among cancer patients undergoing curative intent radiation therapy for head and neck malignancies. Oral Oncol.

[15] Reich M, Leemans CR, Vermorken JB (2014). Best practices in the management of the psychooncologic aspects of head and neck cancer patients: recommendations from the European head and neck cancer society make sense campaign. Ann Oncol.

[16] Molina MA, Cheung MC, Perez EA (2008). African American and poor patients have a dramatically worse prognosis for head and neck cancer: an examination of 20,915 patients. Cancer.

[17] Reitzel LR, Nguyen N, Zafereo ME (2012). Neighborhood deprivation and clinical outcomes among head and neck cancer patients. Heal Place.

[18] Massa ST, Osazuwa-Peters N, Adjei Boakye E (2019). Comparison of the financial burden of survivors of head and neck cancer with other cancer survivors. JAMA Otolaryngol Head Neck Surg.

[19] Penson RT, Fergus LA, Haston RJ (2003). The Kenneth B. Schwartz Center at Massachusetts General Hospital Hematology Oncology Department: Hope for the Homeless. Oncologist.

[20] Park A, Alabaster A, Shen H (2019). Undertreatment of women with locoregionally advanced head and neck cancer. Cancer.

[21] Chang TS, Hou SJ, Su YC (2013). Disparities in oral cancer survival among mentally Ill patients. PLoS ONE.

[22] Satgé D, Nishi M, Culine S, Hennequin M (2012). Awareness on oral cancer in people with intellectual disability. Oral Oncol.

[23] Fagan JJ, Zafereo M, Aswani J (2017). Head and neck surgical subspecialty training in Africa: sustainable models to improve cancer care in developing countries. Head Neck.

[24] De Almeida JR, Byrd JK, Wu R (2014). A systematic review of transoral robotic surgery and radiotherapy for early oropharynx cancer: a systematic review. Laryngoscope.

[25] De Almeida JR, Li R, Magnuson JS (2015). Oncologic outcomes after transoral robotic surgery a multi-institutional study. JAMA Otolaryngol Head Neck Surg.

[26] Steuer CE, El-Deiry M, Parks JR (2017). An update on larynx cancer. CA Cancer J Clin.

[27] Mahmoud O, Sung K, Civantos FJ (2018). Transoral robotic surgery for oropharyngeal squamous cell carcinoma in the era of human papillomavirus. Head Neck.

[28] Salvatori P, Paradisi S, Calabrese L (2014). Sopravvivenza dopo chirurgia ricostruttiva con lembi liberi nel carcinoma spinocellulare del distretto cervico facciale: studio retrospettivo multicentrico. Acta Otorhinolaryngol Ital.

[29] Levendag PC, Teguh DN, Voet P (2007). Dysphagia disorders in patients with cancer of the oropharynx are significantly affected by the radiation therapy dose to the superior and middle constrictor muscle: a dose-effect relationship. Radiother Oncol.

[30] Morisod B, Simon C (2016). Meta-analysis on survival of patients treated with transoral surgery versus radiotherapy for early-stage squamous cell carcinoma of the oropharynx. Head Neck.

[31] Huang SH, Hansen A, Rathod S, O’Sullivan B (2015). Primary surgery versus (chemo)radiotherapy in oropharyngeal cancer: the radiation oncologist’s and medical oncologist’s perspectives. Curr Opin Otolaryngol Head Neck Surg.

[32] Morisod B, Venara-Vulpe II, Alzuphar S (2017). Minimizing adjuvant treatment after transoral robotic surgery through surgical margin revision and exclusion of radiographic extracapsular extension: a prospective observational cohort study. Head Neck.

[33] Olmi P, Crispino S, Fallai C (2003). Locoregionally advanced carcinoma of the oropharynx: conventional radiotherapy vs. accelerated hyperfractionated radiotherapy vs. concomitant radiotherapy and chemotherapy—a multicenter randomized trial. Int J Radiat Oncol Biol Phys.

[34] Von Scotti F, Kapsreiter M, Scherl C (2018). A 9-year analysis of transoral laser microsurgery (TLM) of head and neck cancer on their potential suitability for transoral robotic surgery (TORS) for estimation of future tors-specific caseload. Eur Rev Med Pharmacol Sci.

[35] Thielker J, Grosheva M, Ihrler S (2018). Contemporary management of benign and malignant parotid tumors. Front Surg.

[36] Dulguerov P, Jacobsen MS, Allal AS (2001). Nasal and paranasal sinus carcinoma: are we making progress? A series of 220 patients and a systematic review. Cancer.

[37] Danesh-Sani SA, Sarafraz A, Chamani M, Derakhshandeh H (2016). Paranasal sinuses malignancies: a 12-year review of clinical characteristics. Med Oral Patol Oral Cir Bucal.

[38] Amin MB, Greene FL, Edge SB (2017). The Eighth Edition AJCC Cancer Staging Manual: Continuing to build a bridge from a population-based to a more “personalized” approach to cancer staging. CA Cancer J Clin.

[39] Wuerdemann N, Wittekindt C, Sharma SJ (2017). Risk factors for overall survival outcome in surgically treated human papillomavirus-negative and positive patients with oropharyngeal cancer. Oncol Res Treat.

[40] Huang SH, Xu W, Waldron J (2015). Refining American joint committee on cancer/union for international cancer control TNM stage and prognostic groups for human papillomavirus-related oropharyngeal carcinomas. J Clin Oncol.

[41] Ward DEC, van As-Brooks CJ (2014). Head and neck cancer: treatment, rehabilitation, and outcomes.

[42] Richtlijnendatabase—Hoofd-Hals Tumoren. https://richtlijnendatabase.nl/richtlijn/hoofd-halstumoren/hoofd-halstumoren_-_korte_beschrijving.html

[43] Hoinville L, Taylor C, Zasada M (2019). Improving the effectiveness of cancer multidisciplinary team meetings: analysis of a national survey of MDT members’ opinions about streamlining patient discussions. BMJ Open Qual.

[44] Harris JR, Nesbitt M, Seikaly H (2014). Health care delivery for head-and-neck cancer patients in Alberta: a practice guideline. Curr Oncol.

[45] NICE Improving outcomes in head and neck cancers. https://www.nice.org.uk/guidance/csg6. Accessed 24 Nov 2004

[46] Tsai WC, Kung PT, Wang ST (2015). Beneficial impact of multidisciplinary team management on the survival in different stages of oral cavity cancer patients: results of a nationwide cohort study in Taiwan. Oral Oncol.

[47] Wang YH, Kung PT, Tsai WC (2012). Effects of multidisciplinary care on the survival of patients with oral cavity cancer in Taiwan. Oral Oncol.

[48] Friedland PL, Bozic B, Dewar J (2011). Impact of multidisciplinary team management in head and neck cancer patients. Br J Cancer.

[49] Licitra L, Keilholz U, Tahara M (2016). Evaluation of the benefit and use of multidisciplinary teams in the treatment of head and neck cancer. Oral Oncol.

[50] Badran KW, Heineman TE, Kuan EC, St John MA (2018). Is multidisciplinary team care for head and neck cancer worth it?. Laryngoscope.

[51] Dautremont JF, Rudmik LR, Nakoneshny SC (2016). Understanding the impact of a clinical care pathway for major head and neck cancer resection on postdischarge healthcare utilization. Head Neck.

[52] Chen AY, Callender D, Mansyur C (2000). The impact of clinical pathways on the practice of head and neck oncologic surgery: the University of Texas M.D. Anderson Cancer Center experience. Arch Otolaryngol Head Neck Surg.

[53] Prades J, Remue E, van Hoof E, Borras JM (2015). Is it worth reorganising cancer services on the basis of multidisciplinary teams (MDTs)? A systematic review of the objectives and organisation of MDTs and their impact on patient outcomes. Health Policy (New York).

[54] Messing BP, Ward EC, Lazarus C (2019). Establishing a multidisciplinary head and neck clinical pathway: an implementation evaluation and audit of dysphagia-related services and outcomes. Dysphagia.

[55] Gordon SA, Reiter ER (2016). Effectiveness of critical care pathways for head and neck cancer surgery: a systematic review. Head Neck.

[56] García-Peris P, Parón L, Velasco C (2007). Long-term prevalence of oropharyngeal dysphagia in head and neck cancer patients: impact on quality of life. Clin Nutr.

[57] Govender R, Smith CH, Taylor SA (2017). Swallowing interventions for the treatment of dysphagia after head and neck cancer: a systematic review of behavioural strategies used to promote patient adherence to swallowing exercises. BMC Cancer.

[58] Denaro N, Merlano MC, Russi EG (2013). Dysphagia in head and neck cancer patients: pretreatment evaluation, predictive factors, and assessment during radio-chemotherapy, recommendations. Clin Exp Otorhinolaryngol.

[59] Cock C, Omari T (2017). Diagnosis of swallowing disorders: how we interpret pharyngeal manometry. Curr Gastroenterol Rep.

[60] Elfring T, Boliek CA, Winget M (2014). The relationship between lingual and hypoglossal nerve function and quality of life in head and neck cancer. J Oral Rehabil.

[61] Logemann JA (2014). Critical factors in the oral control needed for chewing and swallowing. J Texture Stud.

[62] Raj P, Gupta DK, Samuel S, Singh SK (2019). Evaluation of swallowing dysfunction in cases of locally advanced squamous cell carcinoma oral cavity pre and post treatment. Int J Otorhinolaryngol Head Neck Surg.

[63] Jafari S, Prince RA, Kim DY, Paydarfar D (2003). Sensory regulation of swallowing and airway protection: a role for the internal superior laryngeal nerve in humans. J Physiol.

[64] Giannitto C, Preda L, Zurlo V (2017). Swallowing disorders after oral cavity and pharyngolaryngeal surgery and role of imaging. Gastroenterol Res Pract.

[65] Pignon JP, le Maître A, Maillard E, Bourhis J (2009). Meta-analysis of chemotherapy in head and neck cancer (MACH-NC): an update on 93 randomised trials and 17,346 patients. Radiother Oncol.

[66] Bentzen J, Toustrup K, Eriksen JG (2015). Locally advanced head and neck cancer treated with accelerated radiotherapy, the hypoxic modifier nimorazole and weekly cisplatin. results from the DAHANCA 18 phase II study. Acta Oncol (Madr).

[67] Christianen MEMC, Verdonck-De Leeuw IM, Doornaert P (2015). Patterns of long-term swallowing dysfunction after definitive radiotherapy or chemoradiation. Radiother Oncol.

[68] Goldsmith T, Jacobson MC (2018). Managing the late effects of chemoradiation on swallowing: Bolstering the beginning, minding the middle, and cocreating the end. Curr Opin Otolaryngol Head Neck Surg.

[69] King SN, Dunlap NE, Tennant PA, Pitts T (2016). Pathophysiology of radiation-induced dysphagia in head and neck cancer. Dysphagia.

[70] Kaae JK, Spejlborg ML, Spork U (2020). Reducing late dysphagia for head and neck cancer survivors with oral gel: a feasibility study. Dysphagia.

[71] Roe JWG, Drinnan MJ, Carding PN (2014). Patient-reported outcomes following parotid-sparing intensity-modulated radiotherapy for head and neck cancer. How important is dysphagia?. Oral Oncol.

[72] Hansen CR, Bertelsen A, Hazell I (2016). Automatic treatment planning improves the clinical quality of head and neck cancer treatment plans. Clin Transl Radiat Oncol.

[73] Petkar I, Bhide S, Newbold K (2017). Dysphagia-optimised Intensity-modulated radiotherapy techniques in pharyngeal cancers: is anyone going to swallow it?. Clin Oncol.

[74] Clarke P, Radford K, Coffey M, Stewart M (2016). Speech and swallow rehabilitation in head and neck cancer: United Kingdom National Multidisciplinary Guidelines. J Laryngol Otol.

[75] Bartella AK, Kamal M, Berman S (2018). Role of swallowing function of tracheotomised patients in major head and neck cancer surgery. J Craniofac Surg.

[76] Leder SB, Joe JK, Ross DA (2005). Presence of a tracheotomy tube and aspiration status in early, postsurgical head and neck cancer patients. Head Neck.

[77] Xin G, Ruohoalho J, Bäck L (2019). Analysis of 255 tracheostomies in an otorhinolaryngology-head and neck surgery tertiary care center: a safe procedure with a wide spectrum of indications. Eur Arch Otorhinolaryngol.

[78] Cheung NH, Napolitano LM (2014). Tracheostomy: epidemiology, indications, timing, technique, and outcomes. Respir Care.

[79] Thompson-Henry S, Braddock B (1995). The modified Evan’s blue dye procedure fails to detect aspiration in the tracheostomized patient: five case reports. Dysphagia.

[80] Béchet S, Hill F, Gilheaney Ó, Walshe M (2016). Diagnostic accuracy of the modified Evan’s blue dye test in detecting aspiration in patients with tracheostomy: a systematic review of the evidence. Dysphagia.

[81] Leder SB, Joe JK, Hill SE, Traube M (2001). Effect of tracheotomy tube occlusion on upper esophageal sphincter and pharyngeal pressures in aspirating and nonaspirating patients. Dysphagia.

[82] Leder SB, Tarro JM, Burrell MI (1996). Effect of occlusion of a tracheotomy tube on aspiration. Dysphagia.

[83] Goff D, Patterson J (2019). Eating and drinking with an inflated tracheostomy cuff: a systematic review of the aspiration risk. Int J Lang Commun Disord.

[84] Ding R, Logemann JA (2005). Swallow physiology in patients with trach cuff inflated or deflated: a retrospective study. Head Neck.

[85] Kang JY, Choi KH, Yun GJ (2012). Does removal of tracheostomy affect dysphagia? A kinematic analysis. Dysphagia.

[86] Terk AR, Leder SB, Burrell MI (2007). Hyoid bone and laryngeal movement dependent upon presence of a tracheotomy tube. Dysphagia.

[87] Leder SB, Ross DA (2010). Confirmation of no causal relationship between tracheotomy and aspiration status: a direct replication study. Dysphagia.

[88] Starmer HH, Gourin CG, Lua LL, Burkhead L (2011). Pretreatment swallowing assessment in head and neck cancer patients. Laryngoscope.

[89] Speyer R (2013). Oropharyngeal dysphagia: screening and assessment. Otolaryngol Clin North Am.

[90] Bours GJJW, Speyer R, Lemmens J (2009). Bedside screening tests vs. videofluoroscopy or fibreoptic endoscopic evaluation of swallowing to detect dysphagia in patients with neurological disorders: systematic review. J Adv Nurs.

[91] Brodsky MB, Suiter DM, González-Fernández M (2016). Screening accuracy for aspiration using bedside water swallow tests: a systematic review and meta-analysis. Chest.

[92] Chen PC, Chuang CH, Leong CP (2016). Systematic review and meta-analysis of the diagnostic accuracy of the water swallow test for screening aspiration in stroke patients. J Adv Nurs.

[93] Daniels SK, Anderson JA, Willson PC (2012). Valid items for screening dysphagia risk in patients with stroke: a systematic review. Stroke.

[94] Kertscher B, Speyer R, Palmieri M, Plant C (2014). Bedside screening to detect oropharyngeal dysphagia in patients with neurological disorders: an updated systematic review. Dysphagia.

[95] Park YH, Bang HL, Han HR, Chang HK (2015). Dysphagia screening measures for use in nursing homes: a systematic review. J Korean Acad Nurs.

[96] Baijens LWJ, Clavé P, Cras P (2016). European society for swallowing disorders—European union geriatric medicine society white paper: oropharyngeal dysphagia as a geriatric syndrome. Clin Interv Aging.

[97] Nund RL, Brown B, Ward EC (2019). What are we really measuring? A content comparison of swallowing outcome measures for head and neck cancer based on the International Classification of Functioning, Disability and Health (ICF). Dysphagia.

[98] Crary MA, Carnaby Mann GD, Groher ME (2005). Initial psychometric assessment of a functional oral intake scale for dysphagia in stroke patients. Arch Phys Med Rehabil.

[99] Vellas B, Villars H, Abellan G (2006). Overview of the MNA®—its history and challenges. J Nutr Heal Aging.

[100] Newman R, Vilardell N, Clavé P, Speyer R (2016). Effect of bolus viscosity on the safety and efficacy of swallowing and the kinematics of the swallow response in patients with oropharyngeal dysphagia: white paper by the European Society for Swallowing Disorders (ESSD). Dysphagia.

[101] American Thoracic Society Quality of Lif Resource. https://qol.thoracic.org/sections/key-concepts/functional-status.html

[102] Ferrans CE, Zerwic JJ, Wilbur JE, Larson JL (2005). Conceptual model of health-related quality of life. J Nurs Scholarsh.

[103] Naik A, John MT, Kohli N (2016). Validation of the English-language version of 5-item Oral Health Impact Profile. J Prosthodont Res.

[104] EORTC Quality of Life Group. https://qol.eortc.org/questionnaires/

[105] Aaronson NK, Ahmedzai S, Bergman B (1993). The European organization for research and treatment of cancer QLQ-C30: a quality-of-life instrument for use in international clinical trials in oncology. J Natl Cancer Inst.

[106] Chen AY, Frankowshi R, Bishop-Leone J (2001). The development and validation of a dysphagia-specific quality-of-life questionnaire for patients with head and neck cancer: the M. D. Anderson Dysphagia Inventory. Arch Otolaryngol Head Neck Surg.

[107] Bjordal K, Hammerlid E, Ahlner-Elmqvist M (1999). Quality of life in head and neck cancer patients: validation of the European Organization for Research and Treatment of Cancer Quality of Life Questionnaire-H and N35. J Clin Oncol.

[108] Mokkink LB, Terwee CB, Patrick DL (2010). The COSMIN study reached international consensus on taxonomy, terminology, and definitions of measurement properties for health-related patient-reported outcomes. J Clin Epidemiol.

[109] Mokkink LB, de Vet HCW, Prinsen CAC (2018). COSMIN Risk of Bias checklist for systematic reviews of Patient-Reported Outcome Measures. Qual Life Res.

[110] Speyer R, Cordier R, Kertscher B, Heijnen BJ (2014). Psychometric properties of questionnaires on functional health status in oropharyngeal dysphagia: a systematic literature review. Biomed Res Int.

[111] Swan K, Cordier R, Brown T, Speyer R (2019). Psychometric properties of visuoperceptual measures of videofluoroscopic and fibre-endoscopic evaluations of swallowing: a systematic review. Dysphagia.

[112] Timmerman AA, Speyer R, Heijnen BJ, Klijn-Zwijnenberg IR (2014). Psychometric characteristics of health-related quality-of-life questionnaires in oropharyngeal dysphagia. Dysphagia.

[113] Edelen MO, Reeve BB (2007). Applying item response theory (IRT) modeling to questionnaire development, evaluation, and refinement. Qual Life Res.

[114] Linacre JM (2016) A user’s guide to Winsteps Raschmodel computer programs: program manual 3.92. 0. Mesa-Press, Chicago

[115] Aryadoust V, Tan HAH, Ng LY (2019). A scientometric review of rasch measurement: the rise and progress of a specialty. Front Psychol.

[116] Cordier R, Joosten A, Clavé P (2017). Evaluating the psychometric properties of the Eating Assessment Tool (EAT-10) using rasch analysis. Dysphagia.

[117] Cordier R, Speyer R, Schindler A (2018). Using rasch analysis to evaluate the reliability and validity of the swallowing quality of life questionnaire: an item response theory approach. Dysphagia.

[118] Kean J, Bisson EF, Brodke DS (2018). An introduction to item response theory and rasch analysis: application using the Eating Assessment Tool (EAT-10). Brain Impair.

[119] Wilmskoetter J, Bonilha H, Hong I (2019). Construct validity of the Eating Assessment Tool (EAT-10). Disabil Rehabil.

[120] COnsensus-based Standards for the selection of health Measurement INstruments. https://www.cosmin.nl

[121] Arrese LC, Carrau R, Plowman EK (2017). Relationship between the Eating Assessment Tool-10 and objective clinical ratings of swallowing function in individuals with head and neck cancer. Dysphagia.

[122] Arrese LC, Schieve HJ, Graham JM (2019). Relationship between oral intake, patient perceived swallowing impairment, and objective videofluoroscopic measures of swallowing in patients with head and neck cancer. Head Neck.

[123] Kendall KA, Kosek SR, Tanner K (2014). Quality-of-life scores compared to objective measures of swallowing after oropharyngeal chemoradiation. Laryngoscope.

[124] Miles A, McMillan J, Ward K, Allen J (2015). Esophageal visualization as an adjunct to the videofluoroscopic study of swallowing. Otolaryngol Neck Surg.

[125] Belafsky PC, Rees CJ, Allen J, Leonard RJ (2010). Pharyngeal dilation in cricopharyngeus muscle dysfunction and Zenker diverticulum. Laryngoscope.

[126] Allen J, Blair D, Miles A (2017). Assessment of videofluoroscopic swallow study findings before and after cricopharyngeal myotomy. Head Neck.

[127] Watts S, Gaziano J, Jacobs J, Richter J (2019). Improving the diagnostic capability of the modified barium swallow study through standardization of an esophageal sweep protocol. Dysphagia.

[128] Bonilha HS, Blair J, Carnes B (2013). Preliminary investigation of the effect of pulse rate on judgments of swallowing impairment and treatment recommendations. Dysphagia.

[129] Hong JY, Hwang NK, Lee G (2020). Radiation safety in videofluoroscopic swallowing study: systematic review. Dysphagia.

[130] McCabe D, Ashford J, Wheeler-Hegland K (2009). Evidence-based systematic review: oropharyngeal dysphagia behavioral treatments. Part IV—Impact of dysphagia treatment on individuals’ postcancer treatments. J Rehabil Res Dev.

[131] Logemann JA, Gensler G, Robbins JA (2008). A randomized study of three interventions for aspiration of thin liquids in patients with dementia or Parkinson’s disease. J Speech Lang Hear Res.

[132] Rosenbek JC, Robbins JA, Roecker EB (1996). A penetration-aspiration scale. Dysphagia.

[133] Rommel N, Borgers C, Van Beckevoort D (2015). Bolus residue scale: an easy-to-use and reliable videofluoroscopic analysis tool to score bolus residue in patients with dysphagia. Int J Otolaryngol.

[134] Pearson WG, Molfenter SM, Smith ZM, Steele CM (2013). Image-based measurement of post-swallow residue: the normalized residue ratio scale. Dysphagia.

[135] Hutcheson KA, Barrow MP, Barringer DA (2017). Dynamic Imaging Grade of Swallowing Toxicity (DIGEST): scale development and validation. Cancer.

[136] Martin-Harris B, Brodsky MB, Michel Y (2008). MBS measurement tool for swallow impairment-MBSimp: establishing a standard. Dysphagia.

[137] Langmore SE, Kenneth SMA, Olsen N (1988). Fiberoptic endoscopic examination of swallowing safety: a new procedure. Dysphagia.

[138] Langmore SE (2017). History of fiberoptic endoscopic evaluation of swallowing for evaluation and management of pharyngeal dysphagia: changes over the years. Dysphagia.

[139] Arens C, Herrmann IF, Rohrbach S (2015). Position paper of the German Society of Oto-Rhino-Laryngology, Head and Neck Surgery and the German Society of Phoniatrics and Pediatric Audiology—current state of clinical and endoscopic diagnostics, evaluation, and therapy of swallowing disorders in c. Laryngorhinootologie.

[140] Nekhlyudov L, Lacchetti C, Davis NB (2017). Head and neck cancer survivorship care guideline: American society of clinical oncology clinical practice guideline endorsement of the American cancer society guideline. J Clin Oncol.

[141] Cocks H, Ah-See K, Capel M, Taylor P (2016). Palliative and supportive care in head and neck cancer: United Kingdom National Multidisciplinary Guidelines. J Laryngol Otol.

[142] Nacci A, Matteucci J, Romeo SO (2016). Complications with fiberoptic endoscopic evaluation of swallowing in 2,820 examinations. Folia Phoniatr Logop.

[143] Farneti D, Schindler A, Fattori B (2018). The role of the audiologist–phoniatrician in performing the dynamic endoscopic study of swallowing. Position statement of the Italian study group on dysphagia (GISD)*. Hear Balanc Commun.

[144] Miller C, Murray J, Pelletier C (2001). Knowledge and skills for speech-language pathologists performing endoscopic assessment. Asha Suppl.

[145] Deutschmann MW, McDonough A, Dort JC (2013). Fiber-optic endoscopic evaluation of swallowing (FEES): predictor of swallowing-related complications in the head and neck cancer population. Head Neck.

[146] Hiss SG, Postma GN (2003). Fiberoptic endoscopic evaluation of swallowing. Laryngoscope.

[147] Langmore SE (2001). Endoscopic evaluation and treatment of swallowing disorders.

[148] Bastian RW (1991). Videoendoscopic evaluation of patients with dysphagia: an adjunct to the modified barium swallow. Otolaryngol Neck Surg.

[149] Fuller SC, Leonard R, Aminpour S, Belafsky PC (2009). Validation of the pharyngeal squeeze maneuver. Otolaryngol Head Neck Surg.

[150] Kaneoka A, Krisciunas GP, Walsh K (2015). A comparison of 2 methods of endoscopic laryngeal sensory testing. Ann Otol Rhinol Laryngol.

[151] Rosenthal DI, Lewin JS, Eisbruch A (2006). Prevention and treatment of dysphagia and aspiration after chemoradiation for head and neck cancer. J Clin Oncol.

[152] Kuo CW, Allen CT, Huang CC, Lee CJ (2017). Murray secretion scale and fiberoptic endoscopic evaluation of swallowing in predicting aspiration in dysphagic patients. Eur Arch Oto-Rhino-Laryngology.

[153] Baijens LWJ, Speyer R, Pilz W, Roodenburg N (2014). FEES protocol derived estimates of sensitivity: aspiration in dysphagic patients. Dysphagia.

[154] Barbon CEA, Steele CM (2015). Efficacy of thickened liquids for eliminating aspiration in head and neck cancer: a systematic review. Otolaryngol Head Neck Surg (United States).

[155] Schindler A, Pizzorni N, Mozzanica F (2016). Functional outcomes after supracricoid laryngectomy: what do we not know and what do we need to know?. Eur Arch Otorhinolaryngol.

[156] Colodny N (2002). Interjudge and intrajudge reliabilities in fiberoptic endoscopic evaluation of swallowing (fees®) using the penetration-aspiration scale: a replication study. Dysphagia.

[157] Neubauer PD, Rademaker AW, Leder SB (2015). The yale pharyngeal residue severity rating scale: an anatomically defined and image-based tool. Dysphagia.

[158] Pizzorni N, Crosetti E, Santambrogio E (2020). The penetration-aspiration scale: adaptation to open partial laryngectomy and reliability analysis. Dysphagia.

[159] Coffey MM, Tolley N, Howard D (2018). An investigation of the post-laryngectomy swallow using videofluoroscopy and fiberoptic endoscopic evaluation of swallowing (FEES). Dysphagia.

[160] Pilz W, Vanbelle S, Kremer B (2016). Observers’ agreement on measurements in fiberoptic endoscopic evaluation of swallowing. Dysphagia.

[161] Langmore SE (2003). Evaluation of oropharyngeal dysphagia: which diagnostic tool is superior?. Curr Opin Otolaryngol Head Neck Surg.

[162] Wu CH, Hsiao TY, Chen JC (1997). Evaluation of swallowing safety with fiberoptic endoscope: comparison with videofluoroscopic technique. Laryngoscope.

[163] Pisegna JM, Langmore SE (2016). Parameters of instrumental swallowing evaluations: describing a diagnostic dilemma. Dysphagia.

[164] Kelly AM, Leslie P, Beale T (2006). Fibreoptic endoscopic evaluation of swallowing and videofluoroscopy: does examination type influence perception of pharyngeal residue severity?. Clin Otolaryngol.

[165] Kelly AM, Drinnan MJ, Leslie P (2007). Assessing penetration and aspiration: how do videofluoroscopy and fiberoptic endoscopic evaluation of swallowing compare?. Laryngoscope.

[166] Scharitzer M, Roesner I, Pokieser P (2019). Simultaneous Radiological and Fiberendoscopic Evaluation of Swallowing (“SIRFES”) in patients after surgery of oropharyngeal/laryngeal cancer and postoperative dysphagia. Dysphagia.

[167] Rommel N, Omari T (2014) Pharyngeal studies. In: Manual of High Resolution Esophageal Manometry. Fox M, Kahrilas P, Pandolfino J, Zerbib F. UNI-MED, Bremen

[168] Ekberg O (2019). Dysphagia—diagnosis and treatment.

[169] Jacob P, Kahrilas PJ, Logemann JA (1989). Upper esophageal sphincter opening and modulation during swallowing. Gastroenterology.

[170] Cook IJ, Dodds WJ, Dantas RO (1989). Opening mechanisms of the human upper esophageal sphincter. Am J Physiol Gastrointest Liver Physiol.

[171] Dantas RO, Kern MK, Massey BT (1990). Effect of swallowed bolus variables on oral and pharyngeal phases of swallowing. Am J Physiol Gastrointest Liver Physiol.

[172] Omari TI, Dejaeger E, Van Beckevoort D (2011). A method to objectively assess swallow function in adults with suspected aspiration. Gastroenterology.

[173] Omari TI, Dejaeger E, Van Beckevoort D (2011). A novel method for the nonradiological assessment of ineffective swallowing. Am J Gastroenterol.

[174] Omari TI, Papathanasopoulos A, Dejaeger E (2011). Reproducibility and agreement of pharyngeal automated impedance manometry with videofluoroscopy. Clin Gastroenterol Hepatol.

[175] Szczesniak MM, Maclean J, Zhang T (2015). Inter-rater reliability and validity of automated impedance manometry analysis and fluoroscopy in dysphagic patients after head and neck cancer radiotherapy. Neurogastroenterol Motil.

[176] Omari TI, Ciucci M, Gozdzikowska K (2020). High-resolution pharyngeal manometry and impedance: protocols and metrics—recommendations of a High-Resolution Pharyngeal Manometry International Working Group. Dysphagia.

[177] Swallow Gateway High Resolution Manometry. https://swallowgateway.com

[178] Albergotti WG, Jordan J, Anthony K (2017). A prospective evaluation of short-term dysphagia after transoral robotic surgery for squamous cell carcinoma of the oropharynx. Cancer.

[179] Patterson JM (2019). Late effects of organ preservation treatment on swallowing and voice; presentation, assessment, and screening. Front Oncol.

[180] Espitalier F, Fanous A, Aviv J (2018). International consensus (ICON) on assessment of oropharyngeal dysphagia. Eur Ann Otorhinolaryngol Head Neck Dis.

[181] Kawai S, Yokota T, Onozawa Y (2017). Risk factors for aspiration pneumonia after definitive chemoradiotherapy or bio-radiotherapy for locally advanced head and neck cancer: a monocentric case control study. BMC Cancer.

[182] Mortensen HR, Jensen K, Grau C (2013). Aspiration pneumonia in patients treated with radiotherapy for head and neck cancer. Acta Oncol (Madr).

[183] Eisbruch A, Lyden T, Bradford CR (2002). Objective assessment of swallowing dysfunction and aspiration after radiation concurrent with chemotherapy for head-and-neck cancer. Int J Radiat Oncol Biol Phys.

[184] Hunter KU, Lee OE, Lyden TH (2014). Aspiration pneumonia after chemo-intensity-modulated radiation therapy of oropharyngeal carcinoma and its clinical and dysphagia-related predictors. Head Neck.

[185] Shirasu H, Yokota T, Hamauchi S (2020). Risk factors for aspiration pneumonia during concurrent chemoradiotherapy or bio-radiotherapy for head and neck cancer. BMC Cancer.

[186] Szczesniak MM, Maclean J, Zhang T (2014). Persistent dysphagia after head and neck radiotherapy: a common and under-reported complication with significant effect on non-cancer-related mortality. Clin Oncol.

[187] Rofes L, Arreola V, Clavé P (2012). The volume-viscosity swallow test for clinical screening of dysphagia and aspiration. Nestle Nutr Inst Workshop Ser.

[188] Fattori B, Giusti P, Mancini V, et al. (2016) Confronto tra videofluoroscopia, endoscopia a fibre ottiche e scintigrafia per la diagnosi di disfagia oro-faringea. Acta Otorhinolaryngol Ital 36:395–402. 10.14639/0392-100X-829 10.14639/0392-100X-829 PMC5225795 27958600

[189] Carnaby GD, Crary MA (2014). Development and validation of a cancer-specific swallowing assessment tool: MASA-C. Support Care Cancer.

[190] Fearon K, Strasser F, Anker SD (2011). Definition and classification of cancer cachexia: an international consensus. Lancet Oncol.

[191] Talbert EE, Guttridge DC (2016). Impaired regeneration: a role for the muscle microenvironment in cancer cachexia. Semin Cell Dev Biol.

[192] Couch ME, Dittus K, Toth MJ (2015). Cancer cachexia update in head and neck cancer: definitions and diagnostic features. Head Neck.

[193] Kwon M, Kim RB, Roh JL (2017). Prevalence and clinical significance of cancer cachexia based on time from treatment in advanced-stage head and neck squamous cell carcinoma. Head Neck.

[194] Grossberg AJ, Chamchod S, Fuller CD (2016). Association of body composition with survival and locoregional control of radiotherapy-treated head and neck squamous cell carcinoma. JAMA Oncol.

[195] Jager-Wittenaar H, Dijkstra PU, Dijkstra G (2017). High prevalence of cachexia in newly diagnosed head and neck cancer patients: an exploratory study. Nutrition.

[196] Matsuzuka T, Kiyota N, Mizusawa J (2019). Clinical impact of cachexia in unresectable locally advanced head and neck cancer: supplementary analysis of a phase II trial (JCOG0706-S2). Jpn J Clin Oncol.

[197] Orell-Kotikangas H, Österlund P, Mäkitie O (2017). Cachexia at diagnosis is associated with poor survival in head and neck cancer patients. Acta Otolaryngol.

[198] Kubrak C, Olson K, Jha N (2010). Nutrition impact symptoms: key determinants of reduced dietary intake, weight loss, and reduced functional capacity of patients with head and neck cancer before treatment. Head Neck.

[199] Schiessel DL, Baracos VE (2018). Barriers to cancer nutrition therapy: excess catabolism of muscle and adipose tissues induced by tumour products and chemotherapy. Proc Nutr Soc.

[200] Butler SG, Stuart A, Leng X (2011). The relationship of aspiration status with tongue and handgrip strength in healthy older adults. J Gerontol Ser A Biol Sci Med Sci.

[201] Maeda K, Akagi J (2016). Sarcopenia is an independent risk factor of dysphagia in hospitalized older people. Geriatr Gerontol Int.

[202] Shiozu H, Higashijima M, Koga T (2015). Association of sarcopenia with swallowing problems, related to nutrition and activities of daily living of elderly individuals. J Phys Ther Sci.

[203] Sporns PB, Muhle P, Hanning U (2017). Atrophy of swallowing muscles is associated with severity of dysphagia and age in patients with acute stroke. J Am Med Dir Assoc.

[204] Wakabayashi H, Matsushima M, Uwano R (2015). Skeletal muscle mass is associated with severe dysphagia in cancer patients. J Cachexia Sarcopenia Muscle.

[205] Murakami K, Hirano H, Watanabe Y (2015). Relationship between swallowing function and the skeletal muscle mass of older adults requiring long-term care. Geriatr Gerontol Int.

[206] Feng X, Todd T, Lintzenich CR (2013). Aging-related geniohyoid muscle atrophy is related to aspiration status in healthy older adults. J Gerontol Ser A Biol Sci Med Sci.

[207] Fujishima I, Fujiu-Kurachi M, Arai H (2019). Sarcopenia and dysphagia: position paper by four professional organizations. Geriatr Gerontol Int.

[208] Macrae PR, Jones RD, Myall DJ (2013). Cross-sectional area of the anterior belly of the digastric muscle: comparison of MRI and ultrasound measures. Dysphagia.

[209] Molfenter SM, Lenell C, Lazarus CL (2019). Volumetric changes to the pharynx in healthy aging: consequence for pharyngeal swallow mechanics and function. Dysphagia.

[210] Sporns KB, Hanning U, Schmidt R (2018). Volumetric assessment of swallowing muscles: a comparison of CT and MRI segmentation. RoFo Fortschritte auf dem Gebiet der Rontgenstrahlen und der Bildgeb Verfahren.

[211] Maeda K, Akagi J (2015). Decreased tongue pressure is associated with sarcopenia and sarcopenic dysphagia in the elderly. Dysphagia.

[212] Maeda K, Ishida Y, Nonogaki T (2020). Development and predictors of sarcopenic dysphagia during hospitalization of older adults. Nutrients.

[213] Ganju RG, Morse R, Hoover A (2019). The impact of sarcopenia on tolerance of radiation and outcome in patients with head and neck cancer receiving chemoradiation. Radiother Oncol.

[214] Wendrich AW, Swartz JE, Bril SI (2017). Low skeletal muscle mass is a predictive factor for chemotherapy dose-limiting toxicity in patients with locally advanced head and neck cancer. Oral Oncol.

[215] Stone L, Olson B, Mowery A (2019). Association between sarcopenia and mortality in patients undergoing surgical excision of head and neck cancer. JAMA Otolaryngol Head Neck Surg.

[216] Shen W, Punyanitya M, Wang ZM (2004). Total body skeletal muscle and adipose tissue volumes: estimation from a single abdominal cross-sectional image. J Appl Physiol.

[217] National Comprehensive Cancer Network (2017) Distress Management. In: NCCN Clin. Pract. Guidel. Oncol. https://www.nccn.org/about/news/ebulletin/ebulletindetail.aspx?ebulletinid=1120

[218] Howren MB, Christensen AJ, Karnell LH, Funk GF (2013). Psychological factors associated with head and neck cancer treatment and survivorship: evidence and opportunities for behavioral medicine. J Consult Clin Psychol.

[219] Björklund M, Sarvimäki A, Berg A (2010). Living with head and neck cancer: a profile of captivity. J Nurs Healthc Chronic Illn.

[220] Longacre ML, Ridge JA, Burtness BA (2012). Psychological functioning of caregivers for head and neck cancer patients. Oral Oncol.

[221] Lang H, France E, Williams B (2013). The psychological experience of living with head and neck cancer: a systematic review and meta-synthesis. Psychooncology.

[222] Massie MJ (2004). Prevalence of depression in patients with cancer. J Natl Cancer Inst Monogr.

[223] Cogwell Anderson RAFK (2012). Psychological and psychosocial implications of head and neck cancer. Internet J Ment Health.

[224] Osazuwa-Peters N, Simpson MC, Zhao L (2018). Suicide risk among cancer survivors: Head and neck versus other cancers. Cancer.

[225] Fingeret MC, Hutcheson KA, Jensen K (2013). Associations among speech, eating, and body image concerns for surgical patients with head and neck cancer. Head Neck.

[226] Chen SC, Yu PJ, Hong MY (2015). Communication dysfunction, body image, and symptom severity in postoperative head and neck cancer patients: factors associated with the amount of speaking after treatment. Support Care Cancer.

[227] Ganzer H, Touger-Decker R, Byham-Gray L (2015). The eating experience after treatment for head and neck cancer: a review of the literature. Oral Oncol.

[228] Bressan V, Bagnasco A, Aleo G (2017). The life experience of nutrition impact symptoms during treatment for head and neck cancer patients: a systematic review and meta-synthesis. Support Care Cancer.

[229] Larsson M, Hedelin B, Johansson I, Athlin E (2006). Eating problems and weight loss for patients with head and neck cancer: a chart review from diagnosis until one year after treatment. Cancer Nurs.

[230] Nund RL, Scarinci NA, Cartmill B (2016). Third-party disability in carers of people with dysphagia following non-surgical management for head and neck cancer. Disabil Rehabil.

[231] Nund RL, Rumbach AF, Debattista BC (2015). Communication changes following non-glottic head and neck cancer management: the perspectives of survivors and carers. Int J Speech Lang Pathol.

[232] Bolt S, Eadie T, Yorkston K (2016). Variables associated with communicative participation after head and neck cancer. JAMA Otolaryngol Head Neck Surg.

[233] Taylor JC, Terrell JE, Ronis DL (2004). Disability in patients with head and neck cancer. Arch Otolaryngol Head Neck Surg.

[234] Koch R, Wittekindt C, Altendorf-Hofmann A (2015). Employment pathways and work-related issues in head and neck cancer survivors. Head Neck.

[235] Jeans C, Ward EC, Cartmill B (2019). Patient perceptions of living with head and neck lymphoedema and the impacts to swallowing, voice and speech function. Eur J Cancer Care (Engl).

[236] Van Wilgen CP, Dijkstra PU, Van Der Laan BFAM (2004). Shoulder and neck morbidity in quality of life after surgery for head and neck cancer. Head Neck.

[237] Semple CJ, Dunwoody L, George Kernohan W (2008). Changes and challenges to patients’ lifestyle patterns following treatment for head and neck cancer. J Adv Nurs.

[238] Penner JL (2009). Psychosocial care of patients with head and neck cancer. Semin Oncol Nurs.

[239] Balfe M, Butow P, O’Sullivan E (2016). The financial impact of head and neck cancer caregiving: a qualitative study. Psychooncology.

[240] Hart SL, Hoyt MA, Diefenbach M (2012). Meta-analysis of efficacy of interventions for elevated depressive symptoms in adults diagnosed with cancer. J Natl Cancer Inst.

[241] Faller H, Schuler M, Richard M (2013). Effects of psycho-oncologic interventions on emotional distress and quality of life in adult patients with cancer: systematic review and meta-analysis. J Clin Oncol.

[242] Holland JC, Reznik I (2005). Pathways for psychosocial care of cancer survivors. Cancer.

[243] Coleman N, Hession N, Connolly A (2011). Psycho-oncology best practice guidelines and a service perspective: conceptualising the fit and towards bridging the gap. Irish J Psychol.

[244] Luckett T, Britton B, Clover K, Rankin NM (2011). Evidence for interventions to improve psychological outcomes in people with head and neck cancer: a systematic review of the literature. Support Care Cancer.

[245] Semple C, Parahoo K, Norman A (2013). Psychosocial interventions for patients with head and neck cancer. Cochrane Database Syst Rev.

[246] Senchak JJ, Fang CY, Bauman JR (2019). Interventions to improve quality of life (QOL) and/or mood in patients with head and neck cancer (HNC): a review of the evidence. Cancers Head Neck.

[247] Humphris G (2016). Psychological management for head and neck cancer patients: United Kingdom National Multidisciplinary Guidelines. J Laryngol Otol.

[248] Haman KL (2008). Psychologic distress and head and neck cancer: Part 1—Review of the literature. J Support Oncol.

[249] Riedl D, Gastl R, Gamper E (2018). Cancer patients’ wish for psychological support during outpatient radiation therapy: findings from a psychooncological monitoring program in clinical routine. Strahlentherapie und Onkol.

[250] Zigmond AS, Snaith RP (1983). The hospital anxiety and depression scale. Acta Psychiatr Scand.

[251] Zabora J, Brintzenhofeszoc K, Jacobsen P (2001). A new psychosocial screening instrument for use with cancer patients. Psychosomatics.

[252] Rogers SN, Semple C, Babb M, Humphris G (2016). Quality of life considerations in head and neck cancer: United Kingdom National Multidisciplinary Guidelines. J Laryngol Otol.

[253] Ojo B, Genden EM, Teng MS (2012). A systematic review of head and neck cancer quality of life assessment instruments. Oral Oncol.

[254] List MA, D’Antonio LL, Cella DF (1996). The performance status scale for head and neck cancer patients and the functional assessment of cancer therapy-head and neck scale: a study of utility and validity. Cancer.

[255] Rosenthal DI, Mendoza TR, Chambers MS (2007). Measuring head and neck cancer symptom burden: the development and validation of the M. D. Anderson symptom inventory, head and neck module. Head Neck.

[256] Marin S, Serra-Prat M, Ortega O, Clavé P (2018). Cost of oropharyngeal dysphagia after stroke: protocol for a systematic review. BMJ Open.

[257] Semenov YR, Starmer HM, Gourin CG (2012). The effect of pneumonia on short-term outcomes and cost of care after head and neck cancer surgery. Laryngoscope.

[258] Genther DJ, Gourin CG (2015). Effect of comorbidity on short-term outcomes and cost of care after head and neck cancer surgery in the elderly. Head Neck.

[259] Gourin CG, Starmer HM, Herbert RJ (2015). Quality of care and short- and long-term outcomes of laryngeal cancer care in the elderly. Laryngoscope.

[260] Attrill S, White S, Murray J (2018). Impact of oropharyngeal dysphagia on healthcare cost and length of stay in hospital: A systematic review. BMC Health Serv Res.

[261] Costa A, Carrión S, Puig-Pey M (2019). Triple adaptation of the mediterranean diet: Design of a meal plan for older people with oropharyngeal dysphagia based on home cooking. Nutrients.

[262] Steele CM, Alsanei WA, Ayanikalath S (2015). The influence of food texture and liquid consistency modification on swallowing physiology and function: a systematic review. Dysphagia.

[263] Gallegos C, Brito-de la Fuente E, Clavé P (2017). Nutritional aspects of dysphagia management. Adv Food Nutr Res.

[264] Son YR, Choi KH, Kim TG (2015). Dysphagia in tongue cancer patients. Ann Rehabil Med.

[265] Ortega O, Bolívar-Prados M, Arreola V (2020). Therapeutic effect, rheological properties and α-amylase resistance of a new mixed starch and xanthan gum thickener on four different phenotypes of patients with oropharyngeal dysphagia. Nutrients.

[266] Bolívar-Prados M, Ibáñez L, Arenas C, Ortega O (2020). 9th ESSD congress, 2019. Dysphagia.

[267] Faulhaber D (2002). National dysphagia diet: standardization for optimal care.

[268] Lam P, Stanschus S, Zaman R, Cichero JA (2017) The International Dysphagia Diet Standardisation Initiative (IDDSI) framework: the Kempen pilot. Br J Neurosci Nurs 13:S18–S26. 10.12968/bjnn.2017.13.sup2.s18

[269] Jun K, Junko F, Risa U, Hiro O, Akiko K, Koji T, Hiroshi Maeda IF (2013) The Classification of Modified Diet for Dysphagic Persons in 2013 in the Japanese Society of Dysphagia Rehabilitation. J Japan Diet Assoc 56:833–839. 10.11379/jjda.56.833

[270] Bolivar-Prados M, Rofes L, Arreola V (2019). Effect of a gum-based thickener on the safety of swallowing in patients with poststroke oropharyngeal dysphagia. Neurogastroenterol Motil.

[271] Sukkar SG, Maggi N, Travalca Cupillo B, Ruggiero C (2018). Optimizing texture modified foods for oro-pharyngeal dysphagia: a difficult but possible target?. Front Nutr.

[272] Høxbroe Michaelsen S, Grønhøj C, Høxbroe Michaelsen J (2017). Quality of life in survivors of oropharyngeal cancer: a systematic review and meta-analysis of 1366 patients. Eur J Cancer.

[273] Martín A, Ortega O, Roca M (2018). Effect of a minimal-massive intervention in hospitalized older patients with oropharyngeal dysphagia: a proof of concept study. J Nutr Heal Aging.

[274] Huckabee ML, McIntosh T, Fuller L (2018). The Test of Masticating and Swallowing Solids (TOMASS): reliability, validity and international normative data. Int J Lang Commun Disord.

[275] Matta Z, Chambers E, Garcia JM, Helverson JMG (2006). Sensory characteristics of beverages prepared with commercial thickeners used for dysphagia diets. J Am Diet Assoc.

[276] Penney B (2014). Use of fluid thickener to reduce dysphagia risk. Nurs Times.

[277] Martin AW (1991). Dietary management of swallowing disorders. Dysphagia.

[278] Shim JS, Oh BM, Han TR (2013). Factors associated with compliance with viscosity-modified diet among dysphagic patients. Ann Rehabil Med.

[279] Easterling C (2017). 25 years of dysphagia rehabilitation: what have we done, what are we doing, and where are we going?. Dysphagia.

[280] Langmore SE, Pisegna JM (2015). Efficacy of exercises to rehabilitate dysphagia: a critique of the literature. Int J Speech Lang Pathol.

[281] Duarte VM, Chhetri DK, Liu YF (2013). Swallow preservation exercises during chemoradiation therapy maintains swallow function. Otolaryngol Head Neck Surg (United States).

[282] Hutcheson KA, Lewin JS (2013). Functional assessment and rehabilitation. How to maximize outcomes. Otolaryngol Clin North Am.

[283] Carnaby-Mann G, Crary MA, Schmalfuss I, Amdur R (2012). “Pharyngocise”: randomized controlled trial of preventative exercises to maintain muscle structure and swallowing function during head-and-neck chemoradiotherapy. Int J Radiat Oncol Biol Phys.

[284] Perry A, Lee SH, Cotton S, Kennedy C (2016). Therapeutic exercises for affecting post-treatment swallowing in people treated for advanced-stage head and neck cancers. Cochrane Database Syst Rev.

[285] Greco E, Simic T, Ringash J (2018). Dysphagia treatment for patients with head and neck cancer undergoing radiation therapy: a meta-analysis review. Int J Radiat Oncol Biol Phys.

[286] Govender R, Smith CH, Barratt H (2020). SIP SMART: A parallel group randomised feasibility trial of a tailored pre-treatment swallowing intervention package compared with usual care for patients with head and neck cancer. BMC Cancer.

[287] Baudelet M, Van Den Steen L, Duprez F (2020). Study protocol for a randomized controlled trial: prophylactic swallowing exercises in head-and-neck cancer patients treated with (chemo)radiotherapy (PRESTO trial). Trials.

[288] Guillen-Sola A, Soler NB, Marco E (2019). Effects of prophylactic swallowing exercises on dysphagia and quality of life in patients with head and neck cancer receiving (chemo) radiotherapy: the Redyor study, a protocol for a randomized clinical trial. Trials.

[289] Ciucci M, Jones CA, Malandraki GA, Hutcheson KA (2016). Dysphagia practice in 2035: beyond fluorography, thickener, and electrical stimulation. Semin Speech Lang.

[290] Crary MA, Carnaby GD, Lagorio LA, Carvajal PJ (2012). Functional and physiological outcomes from an exercise-based dysphagia therapy: a pilot investigation of the mcneill dysphagia therapy program. Arch Phys Med Rehabil.

[291] Lazarus CL, Husaini H, Falciglia D (2014). Effects of exercise on swallowing and tongue strength in patients with oral and oropharyngeal cancer treated with primary radiotherapy with or without chemotherapy. Int J Oral Maxillofac Surg.

[292] Hutcheson KA, Barrow MP, Plowman EK (2018). Expiratory muscle strength training for radiation-associated aspiration after head and neck cancer: a case series. Laryngoscope.

[293] Aarup-Kristensen S, Hansen CR, Forner L (2019). Osteoradionecrosis of the mandible after radiotherapy for head and neck cancer: risk factors and dose-volume correlations. Acta Oncol (Madr).

[294] Strojan P, Hutcheson KA, Eisbruch A (2017). Treatment of late sequelae after radiotherapy for head and neck cancer. Cancer Treat Rev.

[295] Rathy R, Sunil S, Nivia M (2013). Osteoradionecrosis of mandible: case report with review of literature. Contemp Clin Dent.

[296] Nabil S, Samman N (2011). Incidence and prevention of osteoradionecrosis after dental extraction in irradiated patients: a systematic review. Int J Oral Maxillofac Surg.

[297] Thorn JJ, Hansen HS, Specht L, Bastholt L (2000). Osteoradionecrosis of the jaws: clinical characteristics and relation to the field of irradiation. J Oral Maxillofac Surg.

[298] Schuurhuis JM, Stokman MA, Witjes MJH (2018). Patients with advanced periodontal disease before intensity-modulated radiation therapy are prone to develop bone healing problems: a 2-year prospective follow-up study. Support Care Cancer.

[299] Petrovic I, Rosen EB, Matros E (2018). Oral rehabilitation of the cancer patient: a formidable challenge. J Surg Oncol.

[300] Shaw RJ, Butterworth CJ, Silcocks P (2019). HOPON (Hyperbaric Oxygen for the Prevention of Osteoradionecrosis): a randomized controlled trial of hyperbaric oxygen to prevent osteoradionecrosis of the irradiated mandible after dentoalveolar surgery. Int J Radiat Oncol Biol Phys.

[301] Annane D, Depondt J, Aubert P (2004). Hyperbaric oxygen therapy for radionecrosis of the jaw: a randomized, placebo-controlled, double-blind trial from the ORN96 study group. J Clin Oncol.

[302] Beumer J, Marunick M, Esposito S (2011). Maxillofacial rehabilitation: prosthodontic and surgical management of cancer-related, acquired, and congenital defects of the head and neck.

[303] Alberga JM, Vosselman N, Korfage A (2020). What is the optimal timing for implant placement in oral cancer patients?.

[304] Pace-Balzan A, Shaw RJ, Butterworth C (2011). Oral rehabilitation following treatment for oral cancer. Periodontol 2000.

[305] Van Der Maarel-Wierink CD, Vanobbergen JNO, Bronkhorst EM (2013). Oral health care and aspiration pneumonia in frail older people: a systematic literature review. Gerodontology.

[306] Xu B, Boero IJ, Hwang L (2015). Aspiration pneumonia after concurrent chemoradiotherapy for head and neck cancer. Cancer.

[307] Kahn ST, Johnstone PAS (2005). Management of xerostomia related to radiotherapy for head and neck cancer. Oncology (Williston Park).

[308] Valdez IH, Atkinson JC, Ship JA, Fox PC (1993). Major salivary gland function in patients with radiation-induced xerostomia: flow rates and sialochemistry. Int J Radiat Oncol Biol Phys.

[309] Frydrych AM, Slack-Smith LM, Parsons R (2017). Compliance of post-radiation therapy head and neck cancer patients with caries preventive protocols. Aust Dent J.

[310] Ben-David MA, Diamante M, Radawski JD (2007). Lack of osteoradionecrosis of the mandible after intensity-modulated radiotherapy for head and neck cancer: likely contributions of both dental care and improved dose distributions. Int J Radiat Oncol Biol Phys.

[311] Chaveli-López B (2014). Oral toxicity produced by chemotherapy: a systematic review. J Clin Exp Dent.

[312] Owosho AA, Tsai CJ, Lee RS (2017). The prevalence and risk factors associated with osteoradionecrosis of the jaw in oral and oropharyngeal cancer patients treated with intensity-modulated radiation therapy (IMRT): the Memorial Sloan Kettering Cancer Center experience. Oral Oncol.

[313] Studer G, Glanzmann C, Studer SP (2011). Risk-adapted dental care prior to intensity-modulated radiotherapy (IMRT). Schweiz Monatsschr Zahnmed.

[314] Beumer J, Curtis T, Nishimura R (1996) Radiation therapy of head and neck tumors: oral effects, dental manifestations and dental treatment. In: Maxillofacial rehabilitation: prosthodontic and surgical considerations. Ishiyaku EuroAmerica Inc, St. Louis

[315] MacCarthy D, Clarke M, O’Regan M (2017). An audit of the baseline dental status and treatment need of individuals referred to Dublin Dental University Hospital for a pre-radiotherapy dental and oral assessment. J Ir Dent Assoc.

[316] Kumar N et al. (2018) The Oral Management of Oncology Patients Requiring Radiotherapy, Chemotherapy and/or Bone Marrow Transplantation. Clicical Guidelines. The Royal College of Surgeons in England in association with the British Society for Disability and Oral Health. https://www.bsdh.org/index.php/component/edocman/?

[317] Mercadante V, Al Hamad A, Lodi G (2017). Interventions for the management of radiotherapy-induced xerostomia and hyposalivation: a systematic review and meta-analysis. Oral Oncol.

[318] Riley P, Glenny AM, Hua F, Worthington HV (2017). Pharmacological interventions for preventing dry mouth and salivary gland dysfunction following radiotherapy. Cochrane Database Syst Rev.

[319] Hong CHL, Hu S, Haverman T (2018). A systematic review of dental disease management in cancer patients. Support Care Cancer.

[320] Thariat J, Ramus L, Darcourt V (2012). Compliance with fluoride custom trays in irradiated head and neck cancer patients. Support Care Cancer.

[321] Awano S, Ansai T, Takata Y (2008). Oral health and mortality risk from pneumonia in the elderly. J Dent Res.

[322] Berchier CE, Slot DE, Van Der Weijden GA (2010). The efficacy of 0.12% chlorhexidine mouthrinse compared with 0.2% on plaque accumulation and periodontal parameters: a systematic review. J Clin Periodontol.

[323] Meca LB, de Souza FRN, Tanimoto HM (2009). Influence of preventive dental treatment on mutans streptococci counts in patients undergoing head and neck radiotherapy. J Appl Oral Sci.

[324] Gotfredsen K, Walls AWG (2007). What dentition assures oral function?. Clin Oral Implants Res.

[325] Tan H, Peres KG, Peres MA (2016). Retention of teeth and oral health-related quality of life. J Dent Res.

[326] Ortega O, Parra C, Zarcero S (2014). Oral health in older patients with oropharyngeal dysphagia. Age Ageing.

[327] Curtis TA, Cantor R (1974). The forgotten patient in maxillofacial prosthetics. J Prosthet Dent.

[328] Pompa G, Saccucci M, Di Carlo G (2015). Survival of dental implants in patients with oral cancer treated by surgery and radiotherapy: a retrospective study. BMC Oral Health.

[329] Linsen SS, Martini M, Stark H (2012). Long-term results of endosteal implants following radical oral cancer surgery with and without adjuvant radiation therapy. Clin Implant Dent Relat Res.

[330] Butterworth C, McCaul L, Barclay C (2016). Restorative dentistry and oral rehabilitation: United Kingdom National Multidisciplinary Guidelines. J Laryngol Otol.

[331] Korfage A, Schoen PJ, Raghoebar GM (2010). Benefits of dental implants installed during ablative tumour surgery in oral cancer patients: a prospective 5-year clinical trial. Clin Oral Implants Res.

[332] Dijkstra PU, Huisman PM, Roodenburg JLN (2006). Criteria for trismus in head and neck oncology. Int J Oral Maxillofac Surg.

[333] van der Geer SJ, Kamstra JI, Roodenburg JLN (2016). Predictors for trismus in patients receiving radiotherapy. Acta Oncol (Madr).

[334] Owosho AA, Pedreira Ramalho LM, Rosenberg HI (2016). Objective assessment of trismus in oral and oropharyngeal cancer patients treated with intensity-modulated radiation therapy (IMRT). J Cranio-Maxillofacial Surg.

[335] Van Der Molen L, Van Rossum MA, Burkhead LM (2009). Functional outcomes and rehabilitation strategies in patients treated with chemoradiotherapy for advanced head and neck cancer: a systematic review. Eur Arch Otorhinolaryngol.

[336] Hutcheson KA, Bhayani MK, Beadle BM (2013). Eat and exercise during radiotherapy or chemoradiotherapy for pharyngeal cancers: use it or lose it. JAMA Otolaryngol Head Neck Surg.

[337] Rapidis AD, Dijkstra PU, Roodenburg JLN (2015). Trismus in patients with head and neck cancer: etiopathogenesis, diagnosis and management. Clin Otolaryngol.

[338] Bensadoun RJ, Riesenbeck D, Lockhart PB (2010). A systematic review of trismus induced by cancer therapies in head and neck cancer patients. Support Care Cancer.

[339] Gebre-Medhin M, Haghanegi M, Robért L (2016). Dose-volume analysis of radiation-induced trismus in head and neck cancer patients. Acta Oncol (Madr).

[340] Hague C, Beasley W, Garcez K (2018). Prospective evaluation of relationships between radiotherapy dose to masticatory apparatus and trismus. Acta Oncol (Madr).

[341] Aguilar ML, Sandow P, Werning JW (2017). The head and neck cancer patient concern inventory©: patient concerns’ prevalence, dental concerns’ impact, and relationships of concerns with quality of life measures. J Prosthodont.

[342] Carroll WR, Locher JL, Canon CL (2008). Pretreatment swallowing exercises improve swallow function after chemoradiation. Laryngoscope.

[343] Scherpenhuizen A, Van Waes AMA, Janssen LM (2015). The effect of exercise therapy in head and neck cancer patients in the treatment of radiotherapy-induced trismus: a systematic review. Oral Oncol.

[344] Kamstra JI, Dijkstra PU, Van Leeuwen M (2015). Mouth opening in patients irradiated for head and neck cancer: a prospective repeated measures study. Oral Oncol.

[345] Pauli N, Svensson U, Karlsson T, Finizia C (2016). Exercise intervention for the treatment of trismus in head and neck cancer—a prospective two-year follow-up study. Acta Oncol (Madr).

[346] Loh SY, Mcleod RWJ, Elhassan HA (2017). Trismus following different treatment modalities for head and neck cancer: a systematic review of subjective measures. Eur Arch Otorhinolaryngol.

[347] Carvalho APV, Mcneely ML, Vital FMR (2020). Interventions for preventing and treating trismus in patients with head and neck cancer. Cochrane Database Syst Rev.

[348] Montalvo C, Finizia C, Pauli N (2017). Impact of exercise with TheraBite device on trismus and health-related quality of life: a prospective study. Ear Nose Throat J.

[349] Van Der Molen L, Van Rossum MA, Burkhead LM (2011). A randomized preventive rehabilitation trial in advanced head and neck cancer patients treated with chemoradiotherapy: feasibility, compliance, and short-term effects. Dysphagia.

[350] Zatarain LA, Smith DK, Deng J (2018). A randomized feasibility trial to evaluate use of the jaw dynasplint to prevent trismus in patients with head and neck cancer receiving primary or adjuvant radiation-based therapy. Integr Cancer Ther.

[351] Kamstra JI, van Leeuwen M, Roodenburg JLN, Dijkstra PU (2017). Exercise therapy for trismus secondary to head and neck cancer: a systematic review. Head Neck.

[352] Brennan S, Salib O, O’Shea C, Moriarty M (2008). A randomised prospective study of extended tocopherol and pentoxifylline therapy, in addition to carbogen, in the treatment of radiation late effects. Ecancermedicalscience.

[353] Famoso JM, Laughlin B, McBride A, Gonzalez VJ (2018). Pentoxifylline and vitamin E drug compliance after adjuvant breast radiation therapy. Adv Radiat Oncol.

[354] Hartl DM, Cohen M, Juliéron M (2008). Botulinum toxin for radiation-induced facial pain and trismus. Otolaryngol Head Neck Surg.

[355] Bhrany AD, Izzard M, Wood AJ, Futran ND (2007). Coronoidectomy for the treatment of trismus in head and neck cancer patients. Laryngoscope.

[356] Nguyen NP, Sallah S, Karlsson U, Antoine JE (2002). Combined chemotherapy and radiation therapy for head and neck malignancies: quality of life issues. Cancer.

[357] Murphy BA, Gilbert J (2009). Dysphagia in head and neck cancer patients treated with radiation: assessment, sequelae, and rehabilitation. Semin Radiat Oncol.

[358] Servagi-Vernat S, Ali D, Roubieu C (2015). Dysphagia after radiotherapy: state of the art and prevention. Eur Ann Otorhinolaryngol Head Neck Dis.

[359] Moss WJ, Pang J, Orosco RK (2018). Esophageal dilation in head and neck cancer patients: a systematic review and meta-analysis. Laryngoscope.

[360] Francis DO, Weymuller EA, Parvathaneni U (2010). Dysphagia, stricture, and pneumonia in head and neck cancer patients: does treatment modality matter?. Ann Otol Rhinol Laryngol.

[361] McBride SM, Parambi RJ, Jang JW (2014). Intensity-modulated versus conventional radiation therapy for oropharyngeal carcinoma: long-term dysphagia and tumor control outcomes. Head Neck.

[362] Abu-Ghanem S, Sung CK, Junlapan A (2019). Endoscopic management of postradiation dysphagia in head and neck cancer patients: a systematic review. Ann Otol Rhinol Laryngol.

[363] Kocdor P, Siegel ER, Tulunay-Ugur OE (2016). Cricopharyngeal dysfunction: a systematic review comparing outcomes of dilatation, botulinum toxin injection, and myotomy. Laryngoscope.

[364] Knigge MA, Thibeault SL (2018). Swallowing outcomes after cricopharyngeal myotomy: a systematic review. Head Neck.

[365] Federatie Medisch Specialisten; Richtlijnendatabase. Richtlijn Orofaryngeale Dysfagie (2017). https://richtlijnendatabase.nl/richtlijn/orofaryngeale_dysfagie/startpagina_orofaryngeale_dysfagie.html

[366] The Appraisal of Guidelines for Research and Evaluation (AGREE) Enterprise Website. https://www.agreetrust.org/

[367] Scolapio JS, Gostout CJ, Schroeder KW (2001). Dysphagia without endoscopically evident disease: to dilate or not?. Am J Gastroenterol.

[368] Silver N, Gal TJ (2014). Endoscopic CO2 laser management of chemoradiation-related cricopharyngeal stenosis. Ann Otol Rhinol Laryngol.

[369] Dawe N, Patterson J, Hamilton D, Hartley C (2014). Targeted use of endoscopic CO2 laser cricopharyngeal myotomy for improving swallowing function following head and neck cancer treatment. J Laryngol Otol.

[370] Fujimoto Y, Hasegawa Y, Yamada H (2007). Swallowing function following extensive resection of oral or oropharyngeal cancer with laryngeal suspension and cricopharyngeal myotomy. Laryngoscope.

[371] Bird JH, Warner E, Corbridge R (2018). Five-year outcome of endoscopic laser cricopharyngeal myotomy: our experience in ten patients. Clin Otolaryngol.

[372] Campbell BH, Tuominen TC, Toohill RJ (1997). The risk and complications of aspiration following cricopharyngeal myotomy. Am J Med.

[373] Hirano M, Fujita M, Tanaka S, Fujita H (1993). Vocal cord paralysis caused by esophageal cancer surgery. Ann Otol Rhinol Laryngol.

[374] Kos MP, David EFL, Aalders IJ (2008). Long-term results of laryngeal suspension and upper esophageal sphincter myotomy as treatment for life-threatening aspiration. Ann Otol Rhinol Laryngol.

[375] Fujimoto Y, Hasegawa Y, Nakayama B, Matsuura H (1998). Usefulness and limitation of crico-pharyngeal myotomy and laryngeal suspension after wide resection of the tongue or oropharynx. J Otolaryngol Japan.

[376] Lei W, Chai L, Guo J (2013). Modified laryngotracheal separation for intractable aspiration pneumonia in patients with nasopharyngeal carcinoma treated by radiotherapy. Auris Nasus Larynx.

[377] Farlow JL, Birkeland AC, Hardenbergh A (2020). Speech and swallowing outcomes after laryngectomy for the dysfunctional irradiated larynx. Eur Arch Otorhinolaryngol.

[378] Topf MC, Magaña LC, Salmon K (2018). Safety and efficacy of functional laryngectomy for end-stage dysphagia. Laryngoscope.

[379] Hutcheson KA, Alvarez CP, Barringer DA (2012). Outcomes of elective total laryngectomy for laryngopharyngeal dysfunction in disease-free head and neck cancer survivors. Otolaryngol Head Neck Surg (United States).

[380] Weimann A, Braga M, Carli F (2017). ESPEN guideline: clinical nutrition in surgery. Clin Nutr.

[381] Arends J, Bodoky G, Bozzetti F (2006). ESPEN guidelines on enteral nutrition: non-surgical oncology. Clin Nutr.

[382] Trotti A, Bellm LA, Epstein JB (2003). Mucositis incidence, severity and associated outcomes in patients with head and neck cancer receiving radiotherapy with or without chemotherapy: a systematic literature review. Radiother Oncol.

[383] Beijer YJ, Koopman M, Terhaard CHJ (2013). Outcome and toxicity of radiotherapy combined with chemotherapy or cetuximab for head and neck cancer: our experience in one hundred and twenty-five patients. Clin Otolaryngol.

[384] Langenberg M, Terhaard CHJ, Hordijk GJ (2004). Simultaneous radio- and chemotherapy for squamous cell carcinoma of the head and neck in daily clinical practice: 5 Years experience in a University Hospital. Clin Otolaryngol Allied Sci.

[385] Brown TE, Getliffe V, Banks MD (2016). Validation of an updated evidence-based protocol for proactive gastrostomy tube insertion in patients with head and neck cancer. Eur J Clin Nutr.

[386] Karsten RT, Stuiver MM, van der Molen L (2019). From reactive to proactive tube feeding during chemoradiotherapy for head and neck cancer: a clinical prediction model-based approach. Oral Oncol.

[387] Van Der Linden NC, Kok A, Leermakers-Vermeer MJ (2017). Indicators for enteral nutrition use and prophylactic percutaneous endoscopic gastrostomy placement in patients with head and neck cancer undergoing chemoradiotherapy. Nutr Clin Pract.

[388] Wang C, Vainshtein JM, Veksler M (2016). Investigating the clinical significance of body composition changes in patients undergoing chemoradiation for oropharyngeal cancer using analytic morphomics. Springerplus.

[389] Silver HJ, Dietrich MS, Murphy BA (2007). Changes in body mass, energy balance, physical function, and inflammatory state in patients with locally advanced head and neck cancer treated with concurrent chemoradiation after low-dose induction chemotherapy. Head Neck.

[390] Capuano G, Grosso A, Gentile PC (2008). Influence of weight loss on outcomes in patients with head and neck cancer undergoing concomitant chemoradiotherapy. Head Neck.

[391] Paccagnella A, Morello M, Da Mosto MC (2010). Early nutritional intervention improves treatment tolerance and outcomes in head and neck cancer patients undergoing concurrent chemoradiotherapy. Support Care Cancer.

[392] McClelland S, Andrews JZ, Chaudhry H (2018). Prophylactic versus reactive gastrostomy tube placement in advanced head and neck cancer treated with definitive chemoradiotherapy: a systematic review. Oral Oncol.

[393] Nugent B, Lewis S, O’Sullivan JM (2013) Enteral feeding methods for nutritional management in patients with head and neck cancers being treated with radiotherapy and/or chemotherapy. Cochrane Database Syst Rev 31:CD007904. https://doi.org/10.1002/14651858.CD007904.pub3 10.1002/14651858.CD007904.pub3 PMC6769131 23440820

[394] Wang J, Liu M, Liu C (2014). Percutaneous endoscopic gastrostomy versus nasogastric tube feeding for patients with head and neck cancer: a systematic review. J Radiat Res.

[395] Brown TE, Spurgin AL, Ross L (2013). Validated swallowing and nutrition guidelines for patients with head and neck cancer: Identification of high-risk patients for proactive gastrostomy. Head Neck.

[396] Talwar B, Donnelly R, Skelly R, Donaldson M (2016). Nutritional management in head and neck cancer: United Kingdom National Multidisciplinary Guidelines. J Laryngol Otol.

[397] Brown TE, Banks MD, Hughes BGM (2017). Randomised controlled trial of early prophylactic feeding vs standard care in patients with head and neck cancer. Br J Cancer.

[398] Grant DG, Bradley PT, Pothier DD (2009). Complications following gastrostomy tube insertion in patients with head and neck cancer: a prospective multi-institution study, systematic review and meta-analysis. Clin Otolaryngol.

[399] Strijbos D, Keszthelyi D, Bogie RMM (2018). A systematic review and meta-analysis on outcomes and complications of percutaneous endoscopic versus radiologic gastrostomy for enteral feeding. J Clin Gastroenterol.

[400] Madhoun MF, Blankenship MM, Blankenship DM (2011). Prophylactic peg placement in head and neck cancer: how many feeding tubes are unused (and unnecessary)?. World J Gastroenterol.

[401] Taniguchi H, Matsuo K, Nakagawa K (2019). Decline in tongue pressure during perioperative period in cancer patients without oral feeding. Clin Nutr ESPEN.

[402] Prestwich RJD, Murray LJ, Williams GF (2019). Impact of choice of feeding tubes on long-term swallow function following chemoradiotherapy for oropharyngeal carcinoma. Acta Oncol (Madr).

[403] Axelsson L, Silander E, Nyman J (2017). Effect of prophylactic percutaneous endoscopic gastrostomy tube on swallowing in advanced head and neck cancer: a randomized controlled study. Head Neck.

[404] Willemsen ACH, Kok A, van Kuijk SMJ (2020). Prediction model for tube feeding dependency during chemoradiotherapy for at least four weeks in head and neck cancer patients: a tool for prophylactic gastrostomy decision making. Clin Nutr.

[405] von Haehling S, Anker SD (2014). Treatment of cachexia: an overview of recent developments. J Am Med Dir Assoc.

[406] Solís-Martínez O, Plasa-Carvalho V, Phillips-Sixtos G (2018). Effect of eicosapentaenoic acid on body composition and inflammation markers in patients with head and neck squamous cell cancer from a public hospital in Mexico. Nutr Cancer.

[407] Shirai Y, Okugawa Y, Hishida A (2017). Fish oil-enriched nutrition combined with systemic chemotherapy for gastrointestinal cancer patients with cancer cachexia. Sci Rep.

[408] Bairati I, Meyer F, Gélinas M (2005). Randomized trial of antioxidant vitamins to prevent acute adverse effects of radiation therapy in head and neck cancer patients. J Clin Oncol.

[409] Sandmæl JA, Bye A, Solheim TS (2017). Feasibility and preliminary effects of resistance training and nutritional supplements during versus after radiotherapy in patients with head and neck cancer: a pilot randomized trial. Cancer.

[410] Grote M, Maihöfer C, Weigl M (2018). Progressive resistance training in cachectic head and neck cancer patients undergoing radiotherapy: a randomized controlled pilot feasibility trial. Radiat Oncol.

[411] Windholz T, Swanson T, Vanderbyl BL, Jagoe RT (2014). The feasibility and acceptability of neuromuscular electrical stimulation to improve exercise performance in patients with advanced cancer: a pilot study. BMC Palliat Care.

[412] Frowen J, Drosdowsky A, Perry A, Corry J (2016). Long-term swallowing after chemoradiotherapy: prospective study of functional and patient-reported changes over time. Head Neck.

[413] Roa Pauloski B, Logemann JA, Rademaker AW (1993). Speech and swallowing function after anterior tongue and floor of mouth resection with distal flap reconstruction. J Speech Hear Res.

[414] McConnel FMS, Logemann JA, Rademaker AW (1994). Surgical variables affecting postoperative swallowing efficiency in oral cancer patients. Laryngoscope.

[415] Lam L, Samman N (2013). Speech and swallowing following tongue cancer surgery and free flap reconstruction—a systematic review. Oral Oncol.

[416] Arrese LC, Hutcheson KA (2018). Framework for speech-language pathology services in patients with oral cavity and oropharyngeal cancers. Oral Maxillofac Surg Clin North Am.

[417] Manikantan K, Khode S, Sayed SI (2009). Dysphagia in head and neck cancer. Cancer Treat Rev.

[418] Wernick Robinson M, Baiungo J, Hohman M, Hadlock T (2012). Facial rehabilitation. Oper Tech Otolaryngol Head Neck Surg.

[419] Rademaker AW, Logemann JA, Pauloski BR (1993). Recovery of postoperative swallowing in patients undergoing partial laryngectomy. Head Neck.

[420] Eisbruch A, Kim HM, Feng FY (2011). Chemo-IMRT of oropharyngeal cancer aiming to reduce dysphagia: swallowing organs late complication probabilities and dosimetric correlates. Int J Radiat Oncol Biol Phys.

[421] Eisele DW, Yarington CT, Lindeman RC (1988). Indications for the tracheoesophageal diversion procedure and the laryngotracheal separation procedure. Ann Otol Rhinol Laryngol.

[422] Heitmiller RF, Tseng E, Jones B (2000). Prevalence of aspiration and laryngeal penetration in patients with unilateral vocal fold motion impairment. Dysphagia.

[423] Barbu AM, Gniady JP, Vivero RJ (2015). Bedside injection medialization laryngoplasty in immediate postoperative patients. Otolaryngol Head Neck Surg (United States).

[424] Dion GR, Nielsen SW (2019). In-office laryngology injections. Otolaryngol Clin North Am.

[425] Frost M (2001). The role of physical, occupational, and speech therapy in hospice: patient empowerment. Am J Hosp Palliat Med.

[426] Eckman S, Roe J (2005). Speech and language therapists in palliative care: what do we have to offer?. Int J Palliat Nurs.

[427] Balfe DM, Koehler RE, Setzen M (1982). Barium examination of the esophagus after total laryngectomy. Radiology.

[428] Crary MA, Glowasky AL (1996). Using botulinum toxin A to improve speech and swallowing function following total laryngectomy. Arch Otolaryngol Head Neck Surg.

[429] MacLean J, Cotton S, Perry A (2009). Post-laryngectomy: it’s hard to swallow : alian study of prevalence and self-reports of swallowing function after a total laryngectomy. Dysphagia.

[430] Grochowska-Bohatyrewicz E (2009). Dysphagia after total laryngectomy. Otolaryngol Pol.

[431] Maclean J, Szczesniak M, Cotton S (2011). Impact of a laryngectomy and surgical closure technique on swallow biomechanics and dysphagia severity. Otolaryngol Head Neck Surg.

[432] Govender R, Lee MT, Davies TC (2012). Development and preliminary validation of a patient-reported outcome measure for swallowing after total laryngectomy (SOAL questionnaire). Clin Otolaryngol.

[433] Govender R, Lee MT, Drinnan M (2016). Psychometric evaluation of the Swallowing Outcomes after Laryngectomy (SOAL) patient-reported outcome measure. Head Neck.

[434] Terlingen LT, Pilz W, Kuijer M (2018). Diagnosis and treatment of oropharyngeal dysphagia after total laryngectomy with or without pharyngoesophageal reconstruction: systematic review. Head Neck.

[435] Georgiou AM, Kambanaros M (2017). Dysphagia related quality of life (QoL) following total laryngectomy (TL). Int J Disabil Hum Dev.

[436] Lotempio MM, Wang KH, Sadeghi A (2005). Comparison of quality of life outcomes in laryngeal cancer patients following chemoradiation vs. total laryngectomy. Otolaryngol Head Neck Surg.

[437] de Casso C, Slevin NJ, Homer JJ (2008). The impact of radiotherapy on swallowing and speech in patients who undergo total laryngectomy. Otolaryngol Head Neck Surg.

[438] Szuecs M, Kuhnt T, Punke C (2014). Subjective voice quality, communicative ability and swallowing after definitive radio(chemo)therapy, laryngectomy plus radio(chemo)therapy, or organ conservation surgery plus radio(chemo)therapy for laryngeal and hypopharyngeal cancer. J Radiat Res.

[439] Burnip E, Owen SJ, Barker S, Patterson JM (2013). Swallowing outcomes following surgical and non-surgical treatment for advanced laryngeal cancer. J Laryngol Otol.

[440] Robertson SM, Yeo JCL, Dunnet C (2012). Voice, swallowing, and quality of life after total laryngectomy—results of the west of Scotland laryngectomy audit. Head Neck.

[441] Sweeny L, Golden JB, White HN (2012). Incidence and outcomes of stricture formation postlaryngectomy. Otolaryngol Head Neck Surg.

[442] Natt RS, McCormick MS, Clayton JM, Ryall C (2010). Percutaneous chemical myotomy using botulium neurtoxin A under local anaesthesia in the treatment of cricopharyngeal dysphagia following laryngectomy. Auris Nasus Larynx.

[443] Lightbody KA, Wilkie MD, Kinshuck AJ (2015). Injection of botulinum toxin for the treatment of post-laryngectomy pharyngoesophageal spasm-related disorders. Ann R Coll Surg Engl.

[444] Oursin C, Pitzer G, Fournier P (1999). Anterior neopharyngeal pseudodiverticulum: a possible cause of dysphagia in laryngectomized patients. Clin Imaging.

[445] Zhang T, Szczesniak M, Maclean J (2016). Biomechanics of pharyngeal deglutitive function following total laryngectomy. Otolaryngol Head Neck Surg (United States).

[446] Harris RL, Grundy A, Odutoye T (2010). Radiologically guided balloon dilatation of neopharyngeal strictures following total laryngectomy and pharyngolaryngectomy: 21 years’ experience. J Laryngol Otol.

[447] Barrett WL, Gluckman JL, Aron BS (1997). Safety of radiating jejunal interposition grafts in head and neck cancer. Am J Clin Oncol Cancer Clin Trials.

[448] Pitzer G, Oursin C, Wolfensberger M (1998). Anteriores pseudodivertikel nach laryngektomie. HNO.

[449] Athar S (2012). Principles of biomedical ethics.

[450] Latimer EJ (1991). Ethical decision-making in the care of the dying and its applications to clinical practice. J Pain Symptom Manage.

[451] Druml C, Ballmer PE, Druml W (2016). ESPEN guideline on ethical aspects of artificial nutrition and hydration. Clin Nutr.

[452] The Multi-Society Task Force on PVS (1994). Medical aspects of the persistent vegetative state. N Engl J Med.

[453] Checklin M, Bain J, Bath L, Lethbridge K (2020). Patients’ perspectives on what makes a better care experience while undergoing treatment for oropharyngeal dysphagia secondary to head and neck cancer. Dysphagia.

[454] Hu A (2017). Reflections: the value of patient support groups. Otolaryngol Head Neck Surg (United States).

[455] Vakharia KT, Ali MJ, Wang SJ (2007). Quality-of-life impact of participation in a head and neck cancer support group. Otolaryngol Head Neck Surg.

[456] Algtewi E, Owens J, Baker SR (2017). Online support groups for head and neck cancer and health-related quality of life. Qual Life Res.

[457] UK Head and Neck Cancer Patient and Carers Book, Second Edition. https://www.theswallows.org.uk/library-item/uk-patient-carer-book-2nd-edition/

[458] Australian Head and Neck Cancer Patient and Carers Book, Second Edition. https://www.theswallows.org.uk/library-item/australian-2nded/

[459] Spanish Head and Neck Cancer Patient and Carers Book, First Edition. https://www.yumpu.com/es/document/read/62849576/swallows-spanish-spreads

[460] Patterson JM, McColl E, Carding PN (2009). Swallowing performance in patients with head and neck cancer: a simple clinical test. Oral Oncol.

[461] Martino R, Silver F, Teasell R (2009). The toronto bedside swallowing screening test (TOR-BSST) development and validation of a dysphagia screening tool for patients with stroke. Stroke.

[462] Clavé P, Arreola V, Romea M (2008). Accuracy of the volume-viscosity swallow test for clinical screening of oropharyngeal dysphagia and aspiration. Clin Nutr.

[463] Borson S, Scanlan J, Brush M (2000). The mini-cog: a cognitive “vital signs” measure for dementia screening in multi-lingual elderly. Int J Geriatr Psychiatry.

[464] Folstein MF, Folstein SE, McHugh PR (1975). “Mini-mental state”. A practical method for grading the cognitive state of patients for the clinician. J Psychiatr Res.

[465] Elia M (2003) Screening for malnutrition: a multidisciplinary responsibility. Development and use of the ‘Malnutrition Universal Screening Tool’ (‘MUST’) for adults. Malnutrition Advisory Group, a Standing Committee of BAPEN. Redditch: BAPEN. https://www.bapen.org.uk/p

[466] Wilson MMG, Thomas DR, Rubenstein LZ (2005). Appetite assessment: simple appetite questionnaire predicts weight loss in community-dwelling adults and nursing home residents. Am J Clin Nutr.

[467] Jacobson BH, Johnson A, Grywalski C (1997). The Voice Handicap Index (VHI): development and validation. Am J Speech-Language Pathol.

